# Effect of metformin and metformin/linagliptin on gut microbiota in patients with prediabetes

**DOI:** 10.1038/s41598-024-60081-y

**Published:** 2024-04-27

**Authors:** Yoscelina Estrella Martínez-López, Daniel Neri-Rosario, Diego Armando Esquivel-Hernández, Cristian Padron-Manrique, Aarón Vázquez-Jiménez, Jean Paul Sánchez-Castañeda, David Girón-Villalobos, Cristian Mendoza-Ortíz, María de Lourdes Reyes-Escogido, Maria Lola Evia-Viscarra, Alberto Aguilar-Garcia, Osbaldo Resendis-Antonio, Rodolfo Guardado-Mendoza

**Affiliations:** 1 Human Systems Biology Laboratory. Instituto Nacional de Medicina Genómica (INMEGEN), México City, Mexico; 2 Programa de Doctorado en Ciencias Médicas, Odontológicas y de la Salud, Universidad Nacional Autónoma de México (UNAM), Ciudad de México, Mexico; 3 Metabolic Research Laboratory, Department of Medicine and Nutrition, University of Guanajuato, León, Guanajuato, Mexico; 4 Programa de Maestría en Ciencias Bioquímicas, Universidad Nacional Autónoma de México (UNAM), Ciudad de México, Mexico; 5 Programa de Doctorado en Ciencias Biomédicas, Universidad Nacional Autónoma de México (UNAM), Ciudad de México, Mexico; 6 Endocrinology Department Hospital Regional de Alta Especialidad del Bajío, León, Mexico; 7 Coordinación de la Investigación Científica – Red de Apoyo a la Investigación - Centro de Ciencias de la Complejidad, Universidad Nacional Autónoma de México (UNAM), Ciudad de México, Mexico

**Keywords:** Gut microbiota, Metformin, Linagliptin/metformin, Insulin resistance, Pancreatic β-cell function, Systems biology, Endocrinology

## Abstract

Lifestyle modifications, metformin, and linagliptin reduce the incidence of type 2 diabetes (T2D) in people with prediabetes. The gut microbiota (GM) may enhance such interventions' efficacy. We determined the effect of linagliptin/metformin (LM) vs metformin (M) on GM composition and its relationship to insulin sensitivity (IS) and pancreatic β-cell function (Pβf) in patients with prediabetes. A cross-sectional study was conducted at different times: basal, six, and twelve months in 167 Mexican adults with prediabetes. These treatments increased the abundance of GM SCFA-producing bacteria M (*Fusicatenibacter* and *Blautia*) and LM (*Roseburia*, *Bifidobacterium,* and [*Eubacterium*] *hallii group*). We performed a mediation analysis with structural equation models (SEM). In conclusion, M and LM therapies improve insulin sensitivity and Pβf in prediabetics. GM is partially associated with these improvements since the SEM models suggest a weak association between specific bacterial genera and improvements in IS and Pβf.

## Introduction

Prediabetes precedes Type 2 diabetes (T2D) and involves impaired fasting glucose (IFG) and/or impaired glucose tolerance (IGT)^1,2^. Those with prediabetes exhibit insulin resistance (IR), pancreatic β-cell and α-cell dysfunction, and reduced incretin effect^3^. Lifestyle and pharmacological interventions can achieve glycemic control, preventing or delaying T2D and its complications^4–6^.

Lifestyle intervention is the standard treatment for prediabetes, and certain drugs have also demonstrated efficacy in preventing T2D. The combination of linagliptin, metformin, and lifestyle modifications significantly improved glucose metabolism and pancreatic β-cell function (Pβf), reducing T2D incidence in prediabetic individuals compared to metformin and lifestyle intervention^7^. Studies in humans and animals have highlighted the influence of gut microbiota (GM) on the pharmacological effects of metformin and Dipeptidyl Peptidase 4 inhibitors (DPP-4is) (e.g., vildagliptin, sitagliptin, and liraglutide); however, the precise mechanisms by which GM affects the patient's metabolic condition remain unclear^8–13^.

Metformin, a biguanide, is commonly used as a first-line treatment for T2D and prediabetes^14^. Research suggests that metformin reduces GM diversity in murine models of induced T2D while promoting the growth of mucin-degrading bacteria (e.g., *Akkermansia muciniphila*) and SCFA-producing bacteria (e.g., *Roseburia* and *Faecalibacterium prausnitzii*)^9^. The increased abundance of these bacteria is associated with higher levels of goblet cells (mucin-producing), improved glucose tolerance, and reduced proinflammatory interleukin-6 (IL-6)^15^.

On the other hand, linagliptin, a widely used DPP-4i in T2D, is known for its cardiovascular and renal safety. Its efficacy in prediabetes has been demonstrated, improving glucose metabolism and pancreatic islet function^7,16^. Incretin-based therapies utilizing DPP-4is are based on the insulinotropic action of glucagon-like peptide 1 (GLP-1)^17^. By increasing endogenous GLP-1 and insulin levels and reducing glucagon secretion^18^, DPP-4i effectively lowers postprandial blood glucose levels by inhibiting incretin hormone degradation. Although some studies suggest a link between DPP-4is and GM, their exact mechanisms remain unclear.

To evaluate how changes in GM are associated with the clinical response, we assessed the impact of linagliptin/metformin (LM) versus metformin alone (M) on GM composition and its association with insulin sensitivity (Matsuda Index, IS) and pancreatic β-cell function (Pβf) in Mexican patients with prediabetes. Our results indicate that linagliptin/metformin is more clinically effective than metformin alone, and the contribution of GM to the clinical response is relatively low.

## Results

### Participants and clinical outcomes after the intervention

**Figure 1:**
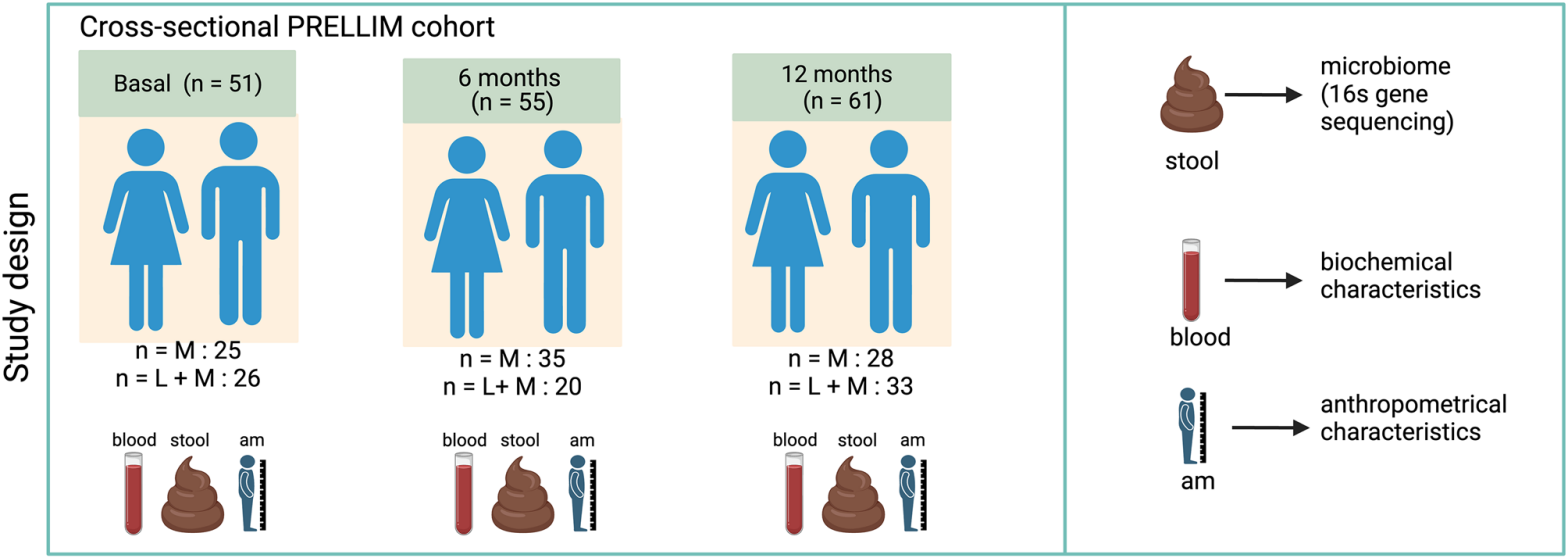
Study profile. This work analyzed the data into two distinct analytical categories: (1) The participants were 51 in the no-treatment group during the baseline evaluation, 55 at the six-month follow-up (with 35 in the M group and 20 in the LM group), and 61 at the 12-month follow-up (comprising 28 in the M group and 33 in the LM group). (2) The gut microbiota samples were 65 in the untreated group (baseline evaluation), 77 at the six-month follow-up (44 in the M group and 33 in the LM group), and 63 at the 12-month follow-up (28 in the group M and 35 in group LM). Created with BioRender.com. LM: The combination of linagliptin + metformin + lifestyle. M: only metformin + lifestyle.

The patients in this study were part of the diabetes prevention trial PRELLIM, a double-blind, randomized parallel clinical trial comparing linagliptin + metformin + lifestyle (LM) to metformin + lifestyle (M) in terms of their effects on glucose metabolism, insulin resistance (IR), and pancreatic islet function [ClinicalTrials.gov ID: NCT03004612 (22/12/2016)]^7^. All participants in PRELLIM were encouraged to participate in the microbiome study. Then, between August 2018 and December 2019, 222 participants were assessed for eligibility and 167 eligible participants were included. These participants were randomly assigned to one of two distinct treatment groups. Specifically, 51 participants were included in the no-treatment group (basal evaluation), 55 at six months follow-up (35 in the M group and 20 in the LM group), and 61 at 12 months follow-up (28 in the M group and 33 in the LM group) (Fig. 1). Unfortunately, not all participants provided a stool sample during follow-up, and some did not complete the prescribed number of follow-up sessions. Accordingly, we organized the data for analysis as a cross-sectional study of groups at different points in time. Specifically, 205 samples were included in the analysis: 65 in the untreated group (baseline evaluation), 77 at the six-month follow-up (44 in the M group and 33 in the LM group), and 63 at the 12-month follow-up (28 in the group M and 35 in group LM). Since the original objective of the study was to observe changes in GM composition over time and its implications on clinical response, we obtained 38 stool samples corresponding to the participants' follow-up at months 6 (n = 12) and 12 (n = 24). Additional results from a dependent contrast analysis are shown in the supplementary material only in the subgroup of 24 participants who completed follow-up at 6 and 12 months (Table S3). Multiple cardio-metabolic risk factors were present in the whole studied population: obesity (51.6%), high total cholesterol (32%), low-HDL (72%), high triglycerides (63.7%), and high blood pressure (25.5%), without any significant difference between groups. None of the patients took medications or supplements affecting glucose metabolism or gut microbiota composition.

### Insulin sensitivity and pancreatic β-cell function improved at six and 12 months of follow-up

**Figure 2:**
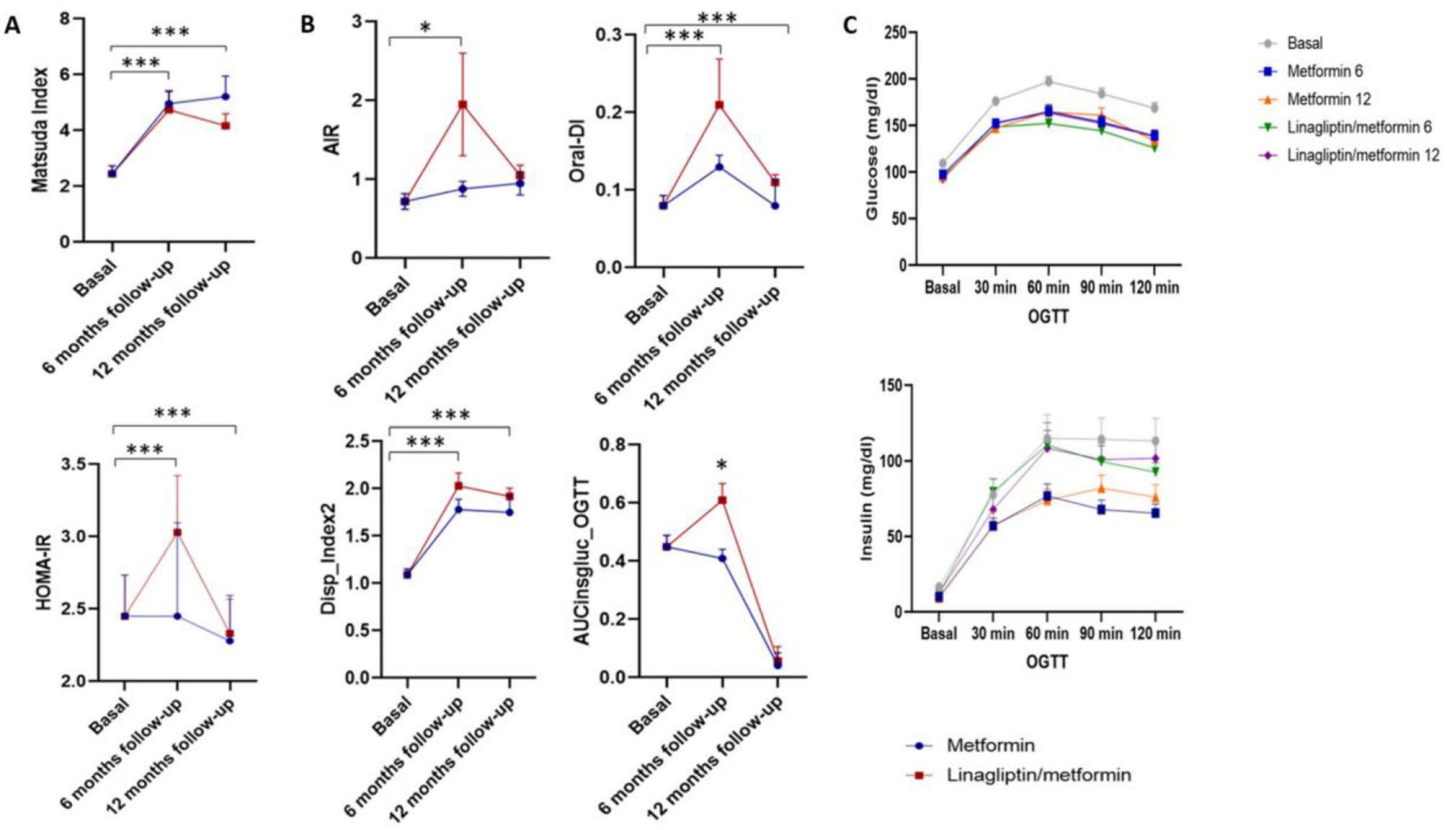
Insulin sensitivity and pancreatic β-cells function. (**A**) IS upper panel; Matsuda index and lower panel; HOMA-IR at baseline, six and 12 months in groups M and LM. (**B**) Basal Pβf, six and 12 months in groups M and LM. Upper left panel; AIR, upper right panel; ORAL-DI, lower left panel; Disp_Index2 and lower right panel; AUCinsgluc_OGTT. (**C**) Glucose and insulin levels during baseline OGTT, six-month and 12-month follow-up: upper panel; glucose and lower panel; insulin. The monthly follow-up was compared with the monthly follow-up. *P < 0.05, **P < 0.01 and ***P < 0.001, one-way ANOVA. *IS* insulin sensitivity, *Pβf* pancreatic β-cells function, *HOMA-IR* Insulin Resistance Index, *AIR* acute insulin response, β-Cell function_2_ (Matsuda*(IncAUCins_0-120_/IncAUCgluc_0-120_)), *Oral_DI* insulin disposition index, *AUCinsgluc_OGTT* glucose area under the curve, *OGTT* curve of oral glucose tolerance.

Age was lower in subjects without pharmacological treatment than at six and 12 months of follow-up (p = 0.0347). Consistent with the original PRELLIM publication, BMI and adiposity showed progressive reductions in this subset of patients at 6 and 12 months. Glucose levels, insulin resistance (IR) index and Pβf significantly improved at six and 12 months of follow-up compared to the baseline group (Table 1) (Tables S1, S2, and S3). Pβf significantly improved in both treatment groups at six months [C-d 1.113 (0.716–1.507), *p* = 0.00001] and 12 months [C-d 1.056 (0.650–1.457), *p* = 0.00001], with a more pronounced improvement in the LM group at both six months [G-Δ 0.3597 (0.189–0.798)] and 12 months [C-d 0.2537 (0.104–0.659)] (Fig. 2 and Table S4).

**Table 1:** Characteristics of the study populations.

Variables	N	Basal	N	Treatment (6 months)	N	Treatment (12 months)	p-value
Age (years)	65	44.08 ± 11.37	77	48.21 ± 9.85ª	63	47.60 ± 12.11^a^	0.0347*
Sex (Femenine %)	65	48(73.85)	77	47(61.04)	63	45(71.43)	0.237
SBP (mmHg)	65	123.90 ± 17.93ª	76	116.45 ± 15.37^b^	61	115.80 ± 15.10^b^	0.0036*
DBP (mmHg)	65	80.90 ± 11.50ª	76	77.02 ± 10.10^b^	61	73.64 ± 10.58^c^	0.0024*
Anthropometric characteristics							
Weight (kg)	65	82.55 ± 18.57ª	77	73.49 ± 14.40^b^	63	73.95 ± 15.02^b^	0.0006*
BMI (kg/m^2^)	65	32.02 ± 6.79ª	77	27.90 ± 4.55^b^	63	28.32 ± 4.35^b^	0.0001*
Waist circumference (cm)	61	97.64 ± 13.58ª	77	89.45 ± 12.80^b^	59	90.63 ± 10.49^b^	0.0004*
Hip circumference (cm)	61	110.57 ± 14.78ª	77	102.70 ± 10.33^b^	59	103.27 ± 10.64^b^	0.0002*
Waist/hip ratio	61	0.88 ± 0.08	77	0.87 ± 0.01	59	0.87 ± 0.08	0.5883
Body fat (%)	64	38.30 ± 8.81ª	77	33.77 ± 8.39^b^	62	34.69 ± 7.49^b^	0.0020*
Visceral fat (AU)	54	9.97 ± 3.99	62	8.76 ± 3.69	58	9.93 ± 3.70	0.2333
Biochemical characteristics							
Fasting glucose (mg/dl)	65	108.47 ± 20.72ª	77	96.05 ± 9.62^b^	63	93.91 ± 9.50^b^	0.0001*
Glucose 120 min (mg/dl)	65	163.58 ± 46.83ª	77	128.60 ± 36.63^b^	63	132.18 ± 33.86^b^	0.0001*
HbA1c (%)	56	5.72 ± 0.79ª	72	5.49 ± 0.39b	60	5.48 ± 0.33^b^	0.0119*
AUC glucose (OGTT)	65	20,569.75 ± 4299.47ª	75	16,872.64 ± 3710.32^b^	61	17,410.17 ± 3023.66^b^	0.0001*
Matsuda Index	51	2.45 ± 2.03ª	64	3.95 ± 3.25^b^	57	3.89 ± 3.02^b^	0.0006*
HOMA-IR	52	4.35 ± 3.94ª	64	2.01 ± 2.93^b^	57	1.90 ± 1.43^b^	0.0001*
QUICKI	51	0.32 ± 0.04ª	64	0.35 ± 0.04^b^	57	0.35 ± 0.04^b^	0.0001*
HOMA-B	51	106.25 ± 98.13	64	93.63 ± 136.27	57	103.96 ± 135.36	0.3251
Insulin basal	51	12.92 ± 10.67ª	64	8.45 ± 12.15^b^	57	8.54 ± 13.05^b^	0.0390*
Insulin 30 minutos (OGTT)	51	60.24 ± 43.47	64	57.06 ± 41.66	56	63.84 ± 48.81	0.7727
Insulin 60 minutos (OGTT)	51	92.52 ± 60.30	64	74.04 ± 70.01	56	79.80 ± 59.00	0.2989
Insulin 90 minutos (OGTT)	51	91.71 ± 60.44ª	64	68.44 ± 58.96^c^	55	82.15 ± 48.51^b^	0.0365*
Insulin 120 minutos (OGTT)	51	91.56 ± 66.55ª	64	65.90 ± 50.08^c^	57	75.71 ± 56.45^b^	0.0120*
AUC insulin (OGTT)	51	9216.52 ± 5431.34ª	64	7546.25 ± 4982.75^b^	57	7738.98 ± 4775.64^b^	0.0013*
AIR *(DI0_30/DG0_30)*	51	0.72 ± 0.70	64	0.95 ± 2.81	57	0.84 ± 0.74	0.1388
Oral_DI (AIR*1/insulin)	51	0.06 ± 0.09	64	0.11 ± 0.26	57	0.10 ± 0.12	0.0623
Insulin secretion *(AUCins0-120/AUCgluc0-120)*	51	0.445 ± 0.28ª	64	0.448 ± 0.28^b^	57	0.444 ± 0.26^b^	0.0020*
β-Cell function_1_ (Matsuda*(AUCins_*0-120*_*/AUCgluc*_*0-120*_*)*	51	2.63 ± 1.20	64	5.18 ± 5.08	56	4.49 ± 5.51	0.1356
β-Cell function_2_ (Matsuda*(IncAUCins_*0-120*_*/IncAUCgluc*_*0-120*_*))*	51	1.09 ± 0.43^b^	64	1.77 ± 0.71ª	57	1.73 ± 0.66ª	0.0001*
Total cholesterol (mg/dl)	62	182.17 ± 35.08	77	182.13 ± 35.93	63	178.71 ± 36.92	0.9864
HDL-c (mg/dl)	61	38.11 ± 11.58^b^	76	43.97 ± 21.87ª	63	43.13 ± 11.82ª	0.0215*
LDL-c (mg/dl)	48	108.09 ± 29.61	38	103.58 ± 42.39	20	106.95 ± 37.43	0.9147
VLDL-c (mg/dl)	47	32.52 ± 13.88	32	33.39 ± 23.09	18	30.04 ± 14.73	0.5294
Triglycerides (mg/dl)	62	163.32 ± 69.82	78	147.20 ± 91.85	63	153.09 ± 120.71	0.4744

*ISBP* systolic blood pressure, *DBP* diastolic blood pressure, *BMI* body mass index, *WC* waist circumference, *AC* hip circumference, *AUC* area under the curve, *IncAUC* increase in area under the curve, *OGTT* curve of oral glucose tolerance, *AIR* acute insulin response, *HOMA-IR* homeostasis model assessment for insulin resistance, *HOMA-B* homeostasis model assessment beta-cell, *Oral_DI* insulin disposition index, *HDL-c* cholesterol high-density lipoprotein cholesterol, *LDL-c* low-density lipoprotein cholesterol, *VLDL-c* very low-density lipoprotein cholesterol, Size effect = Cohen’s D. *p < 0.05.

### Gut microbiome analysis showed a significant impact of hypoglycemic drugs on humans

A total of 17,355,182 quality reads were generated from 203 samples, resulting in 3,713 amplicon sequence variants (ASVs). Individual rarefaction curves indicated high sampling coverage in all samples (Fig. S1). Ecological analysis evaluated α diversity using the non-parametric Kruskal–Wallis test and found no significant changes between groups for Indices Chao 1 (*p* = 0.550) (Fig. S2A), Simpson (*p* = 0.817) (Fig. S2B), Shannon (*p* = 0.796) (Fig. S2C), and Pielou (*p* = 0.469) (Fig. S2D). β diversity analysis with Jaccard distances showed no discernible grouping pattern in microbial diversity between the analysis groups (Fig. S3).

### Spervised machine learning: explainable analysis approach

**Figure 3:**
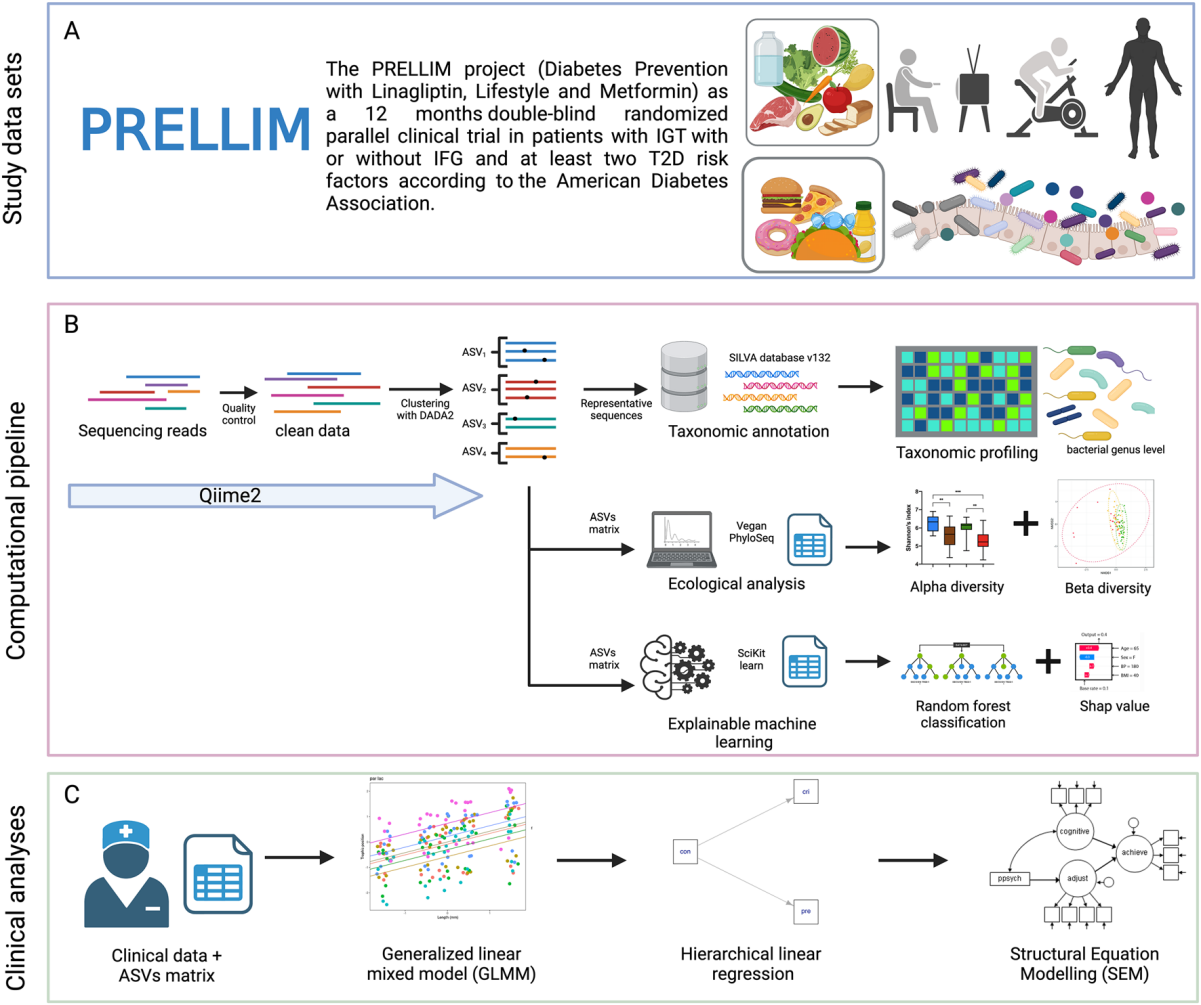
Schematic diagram of the proposed procedure for the data's clinical and GM analysis. It consists of (**A**) PRELLIM data, (**B**) Taxonomic and ecological analysis, and (**C**) Explainable Machine Learning analysis, generalized linear mixed models (GLMM), and structural equation models (SEM). The sequences obtained in the sequencing were processed using the QIIME2 (Quantitative Insights Into Microbial Ecology) analysis platform^19^. The taxonomic classification for end sequence variants was performed using the SILVA database (version 132). The analysis of the GM was performed using the phyloseq object^20^; through this object, the α and β diversity of the study groups in the different months of follow-up was obtained. Subsequently, with the abundance of the other bacterial genera, the genera that classify each pharmacological treatment in the different months of follow-up were obtained using machine learning algorithms (random forest)^21^. Once the bacteria that classified the subjects with M and LM treatment were identified, hierarchical linear regression models were performed to eliminate the effect of confounding variables (obesity, age, and gender). In addition, GLMM and, finally, mediation analysis using SEM. Created with BioRender.com.

**Figure 4:**
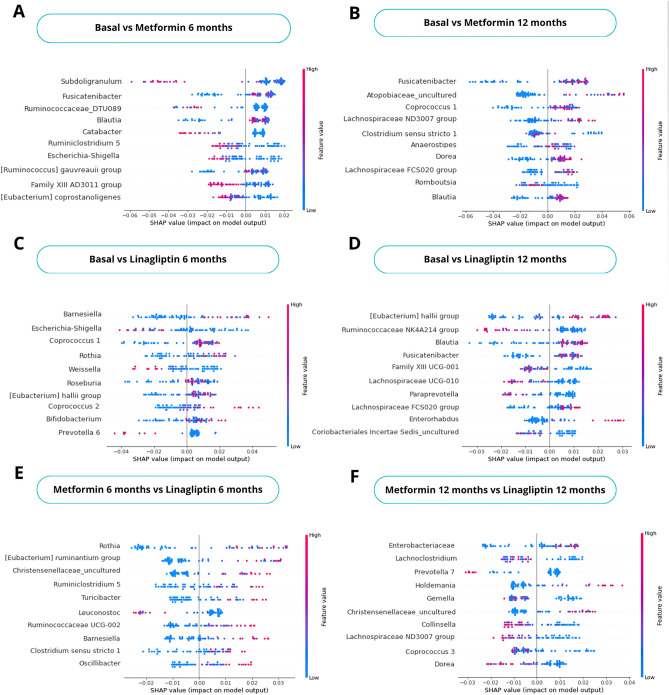
SHAP graph for each hypoglycemic drug and month of follow-up. The figure shows the first ten bacterial genera with the most significant contribution to classifying patients without hypoglycemic drugs (baseline) and those treated with M and LM at six and 12 months of follow-up and between treatments. We have ordered the bacterial genera from most to least relevant from top to bottom. Blue and red represent bacterial genera's low and high abundance, respectively. The higher positive values on the SHAP axis establish the relevance of bacterial genera to classify patients with M or LM at six or 12 months of follow-up, while the negative values establish the relevance of bacterial genera for patients with prediabetes without treatment. (**A**) Baseline vs M with six-month follow-up. (**B**) Baseline vs M with 12-month follow-up. (**C**) Baseline vs LM with six-month follow-up. (**D**) Baseline vs LM with 12-month follow-up. (**E**) M vs LM with a six-month follow-up, and (**F**) M vs LM with a 12-month follow-up.

Using the Random Forest algorithm with post-hoc explanations, we classified different hypoglycemic drugs (LM and M) (Fig. 3). Therapeutic interventions modified GM composition at six and 12 months of follow-up. AUC-ROC (Area Under the Receiver Operating Characteristic Curve) mean values were 0.79 and 0.74 for basal vs. M groups at six and 12 months, respectively (Table S5). Genera *Subdoligranulum, Ruminococcaceae_DTU089, Catabacter, Ruminiclostridium 5*, and *Escherichia-Shigella* identified the basal vs. M group at six months (Fig. 4A). Relevant bacterial genera (*Fusicatenibacter, Blautia,* and *[Ruminococcus] gauvreauii* group), all SCFA producers, identified prediabetes subjects treated for six months with M (Fig. 4A). In contrast, we found eight relevant SCFA-producing bacterial genera (*Fusicatenibacter, Atopobiaceae_uncultured, Coprococcus 1, Lachnospiraceae ND3007 group, Anaerostipes, Dorea, Lachnospiraceae FCS020 group,* and *Blautia*) in patients treated with M for 12 months (Fig. 4B). The effect of M at 12 months suggests cumulative impact, restructuring GM composition and favoring increased SCFA-producing genera abundance^22^.

Next, we compared the basal groups with the LM group at six and 12 months of follow-up; the mean AUC-ROC was 0.6 and 0.66, respectively (Table S5). Seven relevant bacterial genera were identified for classifying subjects with six months of LM treatment: *Barnesiella, Coprococcus 1, Rhotia, Roseburia, [Eubacterium] hallii group, Coprococcus 2*, and *Bifidobacterium. Escherichia-Shigella* classified the basal group and reduced it with hypoglycemic drugs^23^. *[Eubacterium] hallii* group classified subjects treated with LM at both times. F*usicatenibacter, Lachnospiraceae FCS020* group, and *Enterorhabdus* were characteristic in the LM group, all SCFA-producing genera^11,24–26^ (Fig. 4C,D).

On the other hand, we identified bacterial genera in both M and LM intervention groups at six and 12 months of follow-up. At six months, *Rothia, [Eubacterium] Ruminantium* group, *Christensenellaceae_uncultured, Ruminiclostridium 5, Turicibacter, Ruminococcaceae UCG-002, Barnesiella, Clostridium *Sensu Stricto* 1*, and *Oscillibacter* classified the LM group (Fig. 4E). In contrast, at 12 months with LM, *Enterobacteriaceae, Holdemania*, and *Christensenellaceae_uncultured* were characteristic genera (Fig. 4F). The mean AUC-ROC for the M vs LM comparison was 0.66 and 0.74, respectively. Interestingly, we observed that the compositional change produced by LM at six and 12 months is more significant in pathogenic bacteria involved in developing T2D. SCFA-producing genera favor classifying subjects who consumed M, regardless of follow-up month.

### Hypoglycemic drugs and the change in the clinical-metabolic condition explain the increase in insulin sensitivity and pancreatic β-cell function

**Figure 5:**
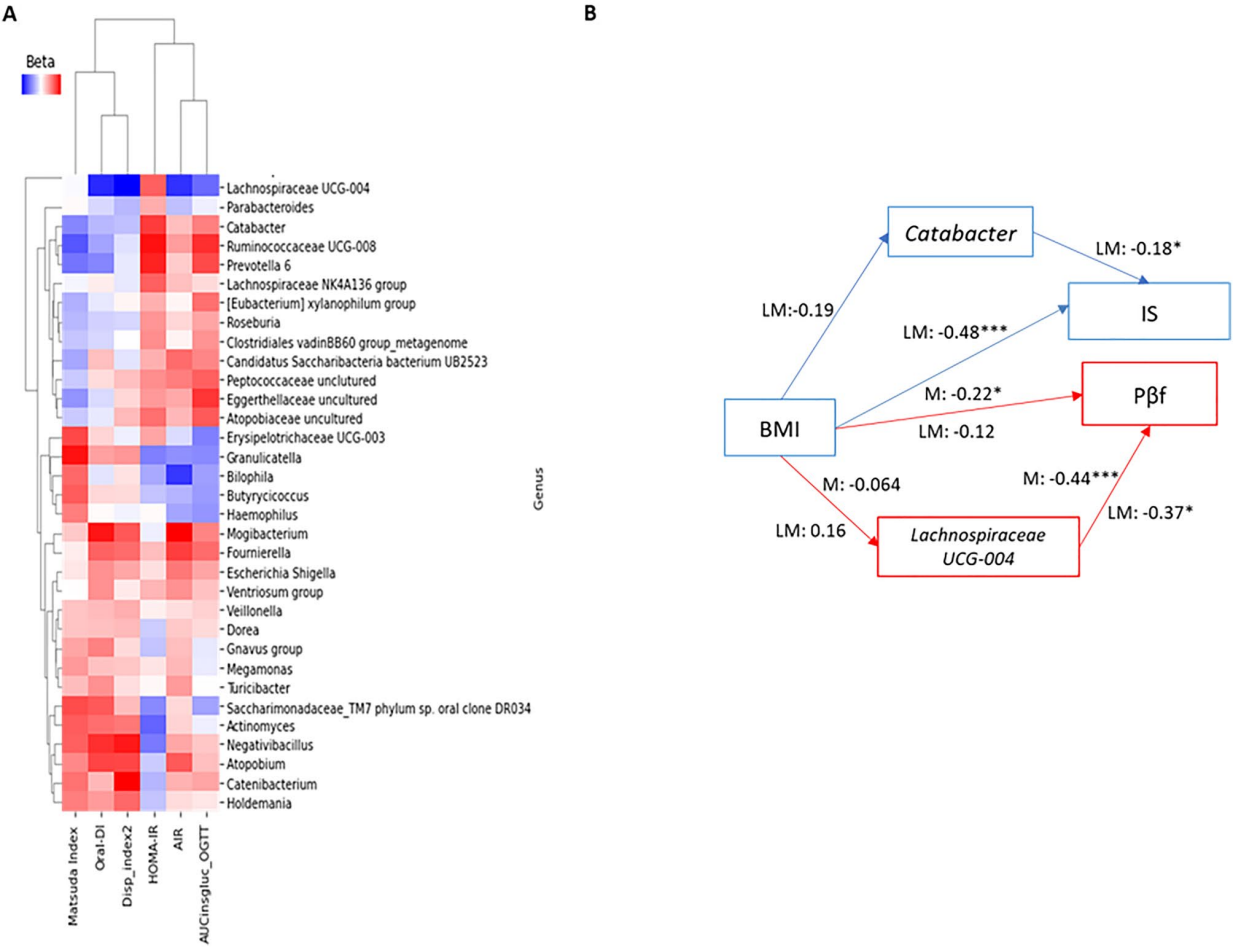
Heatmap resulted from the analysis of GLMM and SEM with standardized path coefficients. (**A**) The color scale represents the β coefficient of the GLMM; when it is red, it means a positive β, and negative when it is blue. All bacteria represented in the heatmap are statistically significant. (**B**) *Catabacter* SEM, the baseline model, showed a relationship between BMI and IS (Matsuda index) and the relationship between BMI and the genus *Catabacter*. *Lachnospiraceae UCG-004* SEM; the baseline model shows a relationship between BMI and IS (Matsuda index) and the relationship between BMI and the genus *Lachnospiraceae UCG-004*. M is a model for metformin treatment, and LM is the model for linagliptin/metformin treatment. **p < 0.05**p < 0.05.

After machine learning analysis and classifying bacteria based on hypoglycemic drugs and months of follow-up, we conducted Generalized Linear Mixed Models (GLMM) considering IS indices and Pβf. Thirty-five significant genera were identified for IS (Matsuda index)^27^: *Roseburi*a (b = − 0.042), *Erysipelotrichaceae UCG-003* (b = 0.048), *Butyrycicoccus* (b = 0.041), *[Eubacterium] xylanophilum* group (b = − 0.045), and *Eggerthellaceae uncultured* (b = − 0.055)^27^. For Pβf (Disp_Index2), significant bacteria included *Dorea* (b = 0.101), *Parabacteroides* (b = − 0.111), *Catenibacterium* (b = 0.233), and *Holdemania* (b = 0.126) (Table 2) (Fig. 5A). We considered a bidirectional relationship between the response variables (IS and Pβf) and bacteria with statistical significance. The abundance of bacterial genera *Butyrycicoccus* (b = 0.666), *[Eubacterium] xylanophilum* group (b = − 0.630), *Granulicatella* (b = 0.544), *Ruminococcaceae UCG-008* (b = − 0.366), *Prevotella 6* (b = − 0.480), and *Catabacter* (b = − 0.508) was modified by the change in IS. Conversely, *Negativibacillus* (b = 0.972) and *Lachnospiraceae UCG-004* (b = − 1.069) were statistically significant for increased Pβf. Models were adjusted for months of follow-up, hypoglycemic drugs, sex, age, BMI, IS, and Pβf (Table S6).

**Table 2:** GLMM for IS, IR, and Pβf indices and effect size and confidence interval for each bacterium.

Fixed effects	β	SE	p-value	95% CI		Eta-Squared	95%CI	
Matsuda Index								
*Roseburia*	− 0.042	0.022	0.050	− 0.084	0.001	0.023	0.0001	0.094
*Erysipelotrichaceae UCG-003*	0.048	0.022	0.031	0.005	0.091	0.021	0.0001	0.089
*Butyrycicoccus*	0.041	0.020	0.044	0.001	0.080	0.027	0.0001	0.099
*[Eubacterium] xylanophilum group*	− 0.045	0.022	0.043	− 0.089	− 0.001	0.029	0.0001	0.104
*Eggerthellaceae uncultured*	− 0.055	0.028	0.049	− 0.109	− 0.001	0.024	0.0001	0.095
HOMA-IR								
*Clostridiales vadinBB60 group_metagenome*	0.061	0.030	0.040	0.003	0.119	0.006	0.0001	0.057
*Lachnospiraceae NK4A136 group*	0.052	0.026	0.049	0.0001	0.103	0.026	0.0001	0.099
*Actinomyces*	− 0.069	0.025	0.005	− 0.118	− 0.021	0.052	0.004	0.139
*Negativibacillus*	− 0.061	0.030	0.043	− 0.119	− 0.002	0.027	0.0001	0.100
AIR								
*Fournierella*	0.067	0.032	0.038	0.004	0.130	0.030	0.0001	0.107
ORAL-DI								
*Gnavus group*	0.050	0.020	0.013	0.011	0.090	0.016	0.0001	0.080
*Turicibacter*	0.035	0.017	0.043	0.001	0.069	0.016	0.0001	0.082
*Mogibacterium*	0.045	0.020	0.022	0.006	0.084	0.063	0.007	0.156
Pβf								
*Dorea*	0.101	0.051	0.049	0.001	0.201	0.005	0.0001	0.053
*Parabacteroides*	− 0.111	0.054	0.040	− 0.218	− 0.005	0.017	0.0001	0.083
*Catenibacterium*	0.233	0.073	0.001	0.090	0.376	0.045	0.002	0.129
*Holdemania*	0.126	0.064	0.049	0.001	0.251	0.021	0.0001	0.089
AUCinsgluc_OGTT								
*Butyricicoccus*	− 0.038	0.019	0.043	− 0.075	− 0.001	0.027	0.0001	0.101
*[Eubacterium] xylanophilum group*	0.041	0.021	0.049	0.001	0.082	0.026	0.0001	0.100
*Atopobiaceae uncultured*	0.048	0.024	0.048	0.001	0.096	0.025	0.0001	0.097
*Peptococcaceae unclutured*	0.046	0.022	0.041	0.002	0.089	0.028	0.0001	0.103
*Lachnospiraceae UCG-004*	− 0.054	0.026	0.038	− 0.105	− 0.003	0.029	0.0001	0.103
N	142

We evaluated the Bacteria X Treatment and Bacteria X Time interaction for IS and Pβf. In the Bacteria X Treatment interaction, we found a significant increase in IS in the LM group associated with *Erysipelotrichaceae UCG-003* (*p* = 0.008). For Pβf, *Erysipelotrichaceae UCG-003* showed statistical significance in the interaction with both LM (*p* = 0.025) and M (*p* = 0.048), while *Lachnospiraceae UCG-004* (*p* = 0.032) and *Turicibacter* (*p* = 0.003) showed significance with M. In the Bacteria X Time interaction, a significant increase in IS at six months was observed with LM treatment for the bacterial genera *Roseburia* (*p* = 0.031), *Erysipelotrichaceae UCG-003* (*p* = 0.018), *Lachnospiraceae NK4A136* (*p* = 0.035), and *Bilophila* (*p* = 0.035). For Pβf, *Erysipelotrichaceae UCG-003* (*p* = 0.004), *Lachnospiraceae NK4A136* (*p* = 0.048), *Granulicatella* (*p* = 0.044), and *Turicibacter* (p = 0.046) showed significance at 6 months, and *Bilophila* (*p* = 0.028) and *Lachnospiraceae UCG-004* (*p* = 0.011) showed significance at 12 months. There was no effect of treatment by time interaction.

Statistically significant bacteria from the GLMM models (with bidirectional effects) were used in subsequent statistical analyses: hierarchical linear regression and structural equations model (SEM). These analyses explored the relationship between GM composition, IS, and Pβf in prediabetes patients treated with hypoglycemic drugs. Hierarchical block models for linear regression revealed that hypoglycemic drugs and BMI significantly explained the increase in IS and Pβf. In the IS model, hypoglycemic drugs contributed a 10% increase (model 1 in Table 3). The addition of gender and age increased the explanation to 14% (model 2 in Table 3), and BMI further increased it to 25% (model 3 in Table 3). However, including the 17 representative genera raised the explanation to 33% (model 4 in Table 3). Among the bacteria analyzed, two were statistically significant: *Catenibacterium* (β = 0.079, *p* < 0.05) and *Catabacter* (β = − 0.083, *p* < 0.05).

**Table 3:** A hierarchical model for the IS (Matsuda Index).

Variable	Model 1	Model 2	Model 3	Model 4
V6 Tmet	0.240***	0.242***	0.147*	0.063
V12 Tmet	0.245***	0.252***	0.204**	0.159*
V6 Tlina + met	0.168**	0.160**	0.106	0.095
V12 Tlina + met	0.169**	0.161**	0.074	0.040
Sex		0.093	0.104*	0.107*
Age		0.360	0.276	0.383*
BMI			− 1.331***	− 1.233***
*Erysypelotrichaceae UCG-00 3*				0.032
*Catanibacterium*				0.079*
*Butyricicoccus*				0.010
*Xylanophilum group*				− 0.038
*Bilophila*				0.025
*Eggerthellaceae_uncultured*				− 0.051
*Actinomyces*				0.020
*Negativibacilis*				0.009
*Mogibacterium*				− 0.007
*Peptococcaceae_uncultured*				− 0.003
*Lachnospiraceae UCG-004*				0.012
*Granulicatella*				0.027
*Fournierella*				0.009
*Ruminococcaceae UCG-008*				− 0.079
*Prevotella 6*				− 0.044
*Atopobium*				− 0.026
*Catabacter*				− 0.083*
Constant	0.391***	− 0.272	1.864**	1.582*
N	166	166	166	166
R2	0.102	0.137	0.249	0.327

*Adjustment for hypoglycemic drugs was set for all models. The intro method was selected, and all block variables were introduced in a single step. Stepwise regression: hypoglycemic drugs in model 1; genera and age in model 2; BMI in model 3; and bacterial genera in model 4.

For Pβf, hypoglycemic drugs explain 22% (model 1 in Table 4). Including gender and age, they reduced the explanation to 21% (model 2 in Table 4), while the addition of BMI explained only 23% (model 3 in Table 4). However, by incorporating the bacterial genera, the variability in the model increased to 35% (model 4 in Table 4). Two bacteria were statistically significant: *Negativibacilis* (β = 0.061, *p* < 0.05) and *Lachnospiraceae UCG-004* (β = − 0.083, *p* < 0.001).

**Table 4:** A hierarchical model for the Pβf. Adjustment for hypoglycemic drugs was set for all models.

Variable	Model 1	Model 2	Model 3	Model 4
V6 Tmet	0.252***	0.256***	0.209**	0.241***
V12 Tmet	0.217***	0.219***	0.196***	0.220***
V6 Tlina + met	0.349***	0.352***	0.325**	0.412***
V12 Tlina + met	0.344***	0.347***	0.304***	0.329***
Sex		0.035	0.041***	0.058
Age		0.036	− 0.005	− 0.058
BMI			− 0.653*	− 0.560
*Erysypelotrichaceae UCG-003*				0.004
*Catanibacterium*				0.034
*Butyricicoccus*				0.006
*Xylanophilum group*				0.007
*Bilophila*				− 0.007
*Eggerthellaceae_uncultured*				− 0.014
*Actinomyces*				0.004
*Negativibacilis*				0.061*
*Mogibacterium*				0.017
*Peptococcaceae_uncultured*				0.028
*Lachnospiraceae UCG-004*				− 0.103**
*Granulicatella*				0.030
*Fournierella*				0.017
*Ruminococcaceae UCG-008*				− 0.072
*Prevotella 6*				0.023
*Atopobium*				0.031
*Catabacter*				− 0.063
Constant	0.299***	0.213	1.261**	1.173*
N	169	169	169	169
R^2^	0.221	0.213	0.234	0.347

The intro method was selected in which all block variables are introduced in a single step. Stepwise regression: hypoglycemic drugs in model 1; gender and age in model 2; BMI in model 3; and bacterial genera in model 4.

### Structural equation modeling (SEM)

To assess the mediating effect of GM on the increase in IS and Pβf in subjects treated with hypoglycemic drugs, we conducted SEM using two significant genera from the GLMM analysis along with a hierarchical model. The SEM also considered BMI to evaluate the impact of weight reduction on clinical variables. *Catabacter* and *Lachnospiraceae UCG-004* were selected as mediator genera for IS and Pβf, respectively, based on the highest beta values and statistical significance obtained in model four of the hierarchical model (see Tables 3 and 4).

In the following SEM, the Matsuda index (IS) is considered the dependent variable, and the genus *Catabacter* is the mediating variable. BMI is strongly associated with the Matsuda index, with significant coefficients in the basal group (b = − 0.24, *p* = 0.022), the M group (b = − 0.27, *p* = 0.170), and the LM group (b = 0.48, *p* = 0.001). However, BMI showed a weak effect on the genus *Catabacter* in all groups (b = 0.23, *p* = 0.917 basal group; b = − 0.14, *p* = 0.411 M group; b = − 0.19, *p* = 0.960 LM group) and a negligible impact on the Matsuda index (b = − 0.27, *p* = 0.062 baseline group; b = − 0.064, *p* = 0.095 M group; b = − 0.18, *p* = 0.510 LM group), except for the LM group, where it was statistically significant (Fig. 5B).

In contrast, we assessed Pβf with the genus *Lachnospiraceae UCG-004* as the mediating variable. BMI had a minor effect on Pβf [BMI—> Pβf (b = − 0.13, *p* = 0.022) in the basal group, (b = − 0.12, *p* = 0.170) for the M group, and (b = 0.22, *p* = 0.001) in the LM group]. BMI did not impact the genus *Lachnospiraceae UCG-004* (b = 0.19 basal group, *p* = − 0.06; b = − 0.64, *p* = 0.411 M group; b = 0.16, *p* = 0.960 LM group). Conversely, *Lachnospiraceae UCG-004* strongly correlated with Pβf (b = 0.057, *p* = 0.062 reference group; b = − 0.44, *p* = 0.095 M group; b = − 0.37, *p* = 0.510 LM group) in the hypoglycemic drug treatment groups, where they were statistically significant (Fig. 5B). These genera have been classified as keystone taxa in other studies with T2D patients^28–30^. These SEMs reveal that bacterial genera partially mediate clinical indices. Conversely, BMI and hypoglycemic drugs are strongly associated with IS and Pβf indices. Additionally, bacterial genera exhibited a weak association with IS and a strong association with Pβf.

To examine the impact of hypoglycemic drugs and the new metabolic condition on bacterial genera abundance, we conducted GLMM (Tweedie distribution) with bacterial genera as the dependent variable and hypoglycemic drugs as the independent variable. The model was adjusted for gender, age, BMI, and the Matsuda index or Pβf. We observed negative effects for *Catabacter* on both IS (β = − 0.503, Eta-Squared = 0.031, *p* = 0.031) (Table S7) and Pβf (β = − 0.990, Eta-Squared = 0.064, *p* = 0.002) (Table S8). These results indicate that the drug-induced changes in clinical indices significantly influence the abundance of the bacteria. It underscores the importance of considering the variation in the metabolic condition to comprehend the complexity of the ecological structure of the intestinal microbiota in subjects with prediabetes.

## Discussion

PRELLIM study was the first randomized clinical trial to evaluate the effect of combined (linagliptin/metformin) therapy on GM in prediabetes subjects with a basal control and metformin monotherapy as a comparator. Consistent with previous reports^7^, at six and 12 months, both drugs significantly improved anthropometric, biochemical, and clinical parameters. The M group increased IS more, while LM favored Pβf. M and LM interventions impacted GM composition at the genus level, resembling findings in T2D. However, novel and specific changes in microbial structure were identified at six and 12 months with both drugs, affecting bacterial genera and ASVs.

The LM intervention impacts GM composition at the genus level, similar to effects observed in animal models using other DPP-4is. Qian Zhang et al. demonstrated vildagliptin's ability to increase butyrate-producing bacteria in rats induced to T2D^31^. Lin Wang et al. explored GM modulation by liraglutide (GLP-1 receptor agonist) and saxagliptin (DPP-4i); they reported increased levels of *Lactobacillus, Allobaculum,* and *Turicibacter* in mice treated with saxagliptin, indicating possible increment in incretins and their effect on glucose homeostasis^32^. Our study results are consistent with an enrichment of butyrate-producing bacteria, including *Lactobacillus, Roseburia, Fusicatenibacter,* and *Blautia*. These genera promote peptide production in the ileum, indirectly reducing hepatic expression of proinflammatory cytokines in T2D^13,33^. Notably, changes in GM composition with each hypoglycemic drug over time differed, particularly in the number of increased SCFA-producing bacteria in each group. Nevertheless, microbial functionality was maintained in each group, a characteristic of GM known as redundancy functions, suggesting species interchangeability within a given microbiota in terms of function^34^.

Current knowledge has established that metformin reduces GM diversity in diabetic mice fed with a high-fat diet, which increases *Akkermansia muciniphila* abundance and other SCFA-producing and mucin-degrading genera^9^. A meta-analysis confirmed the alteration of GM composition by metformin^35^. In our study, Metformin induces GM changes over time in prediabetes patients, partly differing from T2D evidence. At six months, SCFA-producing bacteria (*Fusicatenibacter, Blautia,* and *[Ruminococcus] gauvreauii*) increased, inhibiting enteropathogenic and LPS-producing bacteria (*Proteobacteria, Escherichia-Shigella,* and *Enterococcus*). These differences may result from distinct physiopathological deterioration between prediabetes and T2D^36^.

Interestingly, after a 12-month follow-up, we identified eight abundant SCFA-producing genera: *Fusicatenibacter, Atopobiaceae*, *Coprococcus 1, Lachnospiraceae ND 3007* group, *Anaerostipes, Dorea, Lachnospiraceae FCS020* group, and *Blautia*. Surprisingly, *Akkermansia* and other mucin-degrading bacteria were not significant in subjects with prediabetes, contrary to T2D reports^37–39^. However, the reduction in opportunistic pathogens and T2D-associated genera found in the M-treated group for 12 months aligns with worldwide studies^23,38^. Contrasting both interventions, LM increased bacterial genera, including SCFA producers and opportunistic pathogens, without establishing a clear pattern on the functional redundancy of the GM. Our results suggest that multiple coexisting and taxonomically distinct organisms perform diverse metabolic functions^34,40,41^ (see Fig. 4). We observed that the increase in SCFA-producing genera mediated the change in GM composition.

SCFA-producing genera are crucial for host health. Butyrate is vital to human insulin sensitivity (IS) through incretins. In a high-fat diet mouse model, butyrate supplementation prevents weight gain and increases IS. Butyrate and propionate induce intestinal gluconeogenesis, improving peripheral glucose production and IS^42^. GM changes influence the gut metabolome, affecting butyrate and acetate production^43^, key gut-derived metabolites in insulin resistance (IR) and glycemic control. Increased intestinal gluconeogenesis from these SCFAs in rodents reduces hepatic gluconeogenesis, appetite, and weight, leading to better glucose homeostasis^44^.

Most studies focus on short-term GM and glucose homeostasis associations. Moreover, the impact of hypoglycemic drugs on GM changes and metabolic improvement remains unclear^45^. Our findings show low associations (r = 0.1–0.2) between bacterial abundance of specific genera and clinical variables (fasting glucose, postprandial glucose, glucose AUC, HOMA-RI, or Matsuda Index) in diverse populations and study models. However, hypoglycemic drugs affect GM structure, anthropometric (reduced weight, body fat, and waist circumference), biochemical (increased IS and Pβf), and clinical (lower systolic and diastolic blood pressure) parameters, influencing the associations. In our study, hypoglycemic drugs improved clinical indices with a low GM contribution, as previously reported^46^. Using SEM, we found a strong relationship between BMI (not total adiposity) with IS and Pβf. Metformin and linagliptin effectively reduced weight and fat percentage in overweight and obese insulin-resistant outpatients^47^. Weight loss was the primary predictor of improved IS, while weight regain predicted reduced IS. Weight loss maintenance programs are crucial for preserving metabolic benefits. Physical activity and a balanced diet increase IS in patients with obesity and T2D^48^.

On the other hand, the bacterial abundance of SCFA-producing genera weakly explained the changes in IS and Pβf, with an eta squared of 0.01 in both cases, compared to the effects of hypoglycemic drugs and weight loss (See Table S6 and S7). Thus, we conclude that hypoglycemic drugs strongly impact metabolic conditions (IS and secretion) and moderately influence GM's composition. On the other hand, GM has a lower effect on metabolic changes. To reinforce this, we evaluated bacterial genera abundance using a GLMM, adjusting for hypoglycemic drugs, change in IS, and increased Pβf. We found that the shift in metabolic condition modifies the GM's structure.

Recent findings suggest that gut dysbiosis is linked to metabolic diseases like obesity, diabetes, and non-alcoholic fatty liver disease^49^. These discoveries support the coevolution theory between humans and the GM, profoundly affecting various host responses. It is clear that multiple variables influence glucose metabolism in prediabetes and diabetes prevention, and it cannot be explained by only one factor; in this context, GM seems to play a limited role, which still has to be elucidated in more detail. A limitation of our study is that we only measured GM composition and didn't analyze metabolites or other microbiota functionality measures. Nonetheless, our study provides the first evaluation of the effect of DPP-4 inhibitors on GM composition in humans with prediabetes.

## Conclusions

Our study reveals that changes in GM have a low impact in mediating the effect of lifestyle, metformin, and linagliptin/metformin on glucose metabolism, IS, and Pβf in individuals with prediabetes. Despite the observed increase in SCFA-producing bacteria in the GM following these treatments, the SEM suggests a weak association between specific bacterial genera and improvements in IS and Pβf. Therefore, the primary mechanism of metabolic improvement in prediabetic patients is more directly attributable to the pharmacological effects of the hypoglycemic drugs with only a partial modulation of the GM. Future omics studies with long-term follow-up will determine the extent of drugs' hypoglycemic effect via GM modifications and its role in T2D development and progression.

## Methods

### Trial design and oversight

This study was part of a randomized, double-blind, placebo-controlled clinical trial [ClinicalTrials.gov ID: NCT03004612 (22/12/2016)]. Participants were enrolled between August 2018 and December 2019 as part of the PRELLIM project7 (Prevention of diabetes with linagliptin, lifestyle, and metformin). Further details are in the PRELLIM article^7^.

### Participants and intervention procedure

Eligible participants with prediabetes (per ADA criteria) and no prior glycemic medication were randomly assigned to two groups in a 1:1 ratio: (i) Linagliptin + metformin + lifestyle (LM group): patients started on linagliptin/metformin 2.5/850 mg once daily for a month, then increased to twice daily until study end. (ii) Metformin + lifestyle (group M): patients began with 850 mg metformin once daily, then increased to twice daily. Identical envelopes contained metformin 850 mg and linagliptin/metformin 2.5/850 mg. Both groups received the same lifestyle program. Monthly follow-up visits assessed adherence and side effects and included nutritional evaluation. OGTTs were done at baseline, six, and 12 months. Primary outcomes were changes in the GM composition; Glucose levels, insulin resistance, and pancreatic β-cell function were secondary outcomes^7^.

### The detailed inclusion criteria

167 patients were screened with anthropometric, nutritional, biochemical, and metabolic evaluation, including oral glucose tolerance test and hyperglycemic clamp at the Metabolic Research Laboratory, Hospital Regional de Alta Especialidad del Bajío. Patients were eligible for enrollment in the study based on the following criteria: (i) IGT (two h glucose levels 140–199 mg/dL) during oral glucose tolerance test, ± IFG (fasting glucose 100–125 mg/dL); (ii) age 18–65 years; (iii) ≥ 2 T2D risk factors per ADA^50^.

Exclusion criteria: (i) glucose-affecting treatments in past three months; (ii) glucose/metabolism-related conditions; (iii) recent use of antibiotics, proton pump inhibitors, or pharmaceutical-grade probiotics; (iv) fasting plasma triglycerides > 400 mg/dL; (v) pregnancy; (vi) systolic blood pressure > 180 mm Hg or diastolic > 105 mm Hg; (vii) glucose homeostasis-affecting meds/conditions (e.g., thiazides, beta-blockers, glucocorticoids, weight-reducing drugs, Cushing's syndrome, thyrotoxicosis).

To carry out this research, the ethical standards, the Regulations of the General Health Law on Research for Health, and the Declaration of Helsinki of the World Medical Association of the 52nd General Assembly, Edinburgh, Scotland, October 2000 have been considered with clarification note on paragraph 29 added by the General Assembly, Washington 2002 and current international codes and standards of good clinical research practice. Written informed consent was obtained from all participants before enrollment in this study. The Research and Ethical Committee approved the study at the Hospital Regional de Alta Especialidad del Bajío (CI-HRAEB-2017-048 and CEI-22-16 extension), registered with the Mexico Secretary of Health.

### Anthropometric measures

Weight and body composition were assessed via the Tanita SC-240 Scale: Monthly weight recording and bioimpedance every six months in fasting conditions. Total body fat was measured in %, and visceral fat was measured in arbitrary units^7^.

### Oral glucose tolerance test (OGTT)

Subjects arrived at the University of Guanajuato's Metabolic Research Laboratory between 7 and 8 a.m., fasting. An intravenous catheter was placed, and the first blood sample was drawn. Next, they ingested 75 g of glucose. Serum samples for glucose and insulin measurement were drawn at − 15 and 0 min and every 30 min afterward for two hours, with 4 ml of blood taken each time^7^.

### Measurements

Glucose was measured with Analox glucoanalyzer GM9 and colorimetric glucose oxidase (Vitros 5600; Ortho Clinical Diagnostics). Lipid levels were measured by dry chemistry with colorimetric method (Vitros 5600; Ortho Clinical Diagnostics). Insulin (μU/ml) and C-peptide (ng/ml) were measured by chemiluminescent immunometric assay (IMMULITE 2000 Immunoassay system, Siemens). HbA1c was determined using high-performance liquid chromatography with DS-5 Analyzer (Drew Scientific, Inc. Miami, FL, USA). Personnel performing measurements were blinded to treatment allocation^7^.

### Calculations

OGTT measurements: Incremental and AUC calculated via trapezoidal rule. Insulin secretion derived from AUCinsulin_0_120_ (μU/ml) divided by AUCglucose_0_120_ (mg/dl) during OGTT. Pβf measurements obtained: (i) AIR calculated as insulin change (μU/ml) from 0 to 30 min divided by glucose change (mg/dl) from 0 to 30 min during OGTT; (ii) IS/IR index (DI) during OGTT calculated as (AUCinsulin_0_120_ (μU/ml)/AUCglucose_0_120_ (mg/dl))*Matsuda index and (IncAUCinsulin0_120 (μU/ml)/IncAUCglucose0_120 (mg/dl))*Matsuda index; (iii) Oral disposition index calculated as the product of AIR and 1/fasting insulin (μU/ml). Insulin sensitivity during OGTT calculated with Matsuda index (10,000 √[(Glucosefasting (mg/dl) insulinfasting (μU/ml)) × (Glucosemean × Insulinmean)]) and fasting with homeostasis model assessment (HOMA-IR = fasting insulin (μU/ml) × fasting glucose (mg/dl)/405)^51^.

### Randomization and masking

Patients were randomly assigned in a 1:1 ratio to receive a fixed combination of linagliptin/metformin 2.5/850 mg every 12 h + lifestyle modification program or metformin pills of 850 mg every 12 h + lifestyle modification program. Randomization was performed by a nutritionist who was not involved in the patient's follow-up using an electronic random numbers assignment system. Participants and investigators involved in the patient’s follow-up and outcome measurements were masked to treatment allocation during the entire study using identical envelopes for pills^7^.

### Interventions

(i) Linagliptin + metformin + lifestyle (LM group): Patients allocated to this group started fixed combination pills of linagliptin/metformin 2.5/850 mg once daily during the first month, and after that, the dose was increased to 2.5/850 mg twice daily until the end of the study. (ii) Metformin + lifestyle (M group): Patients in this group started taking metformin pills of 850 mg once daily during the first month and increased to 850 mg twice daily until the end of the study. Pills of metformin 850 mg and linagliptin/metformin 2.5/850 mg were prepared using identical envelopes. Both groups received the same lifestyle implementation program based on a prescribed diet to reduce their body weight by at least 5–7%, adjusting their energy requirements based on their weight, and composed from 55 to 60% of carbohydrates, 25–30% fat, and 10–15% proteins. Patients were advised to start with 45 min/week of mild-moderate exercise and increase the duration and frequency or intensity of exercise every two weeks until reaching 150 min/week of moderate activity or 75 min/week of intense activity^7^.

### Fecal sample collection and processing protocol

Fecal samples from intervention and control groups were collected in sterile containers at zero, six, and twelve months. Samples were homogenized and stored at  − 80 °C in sterile 1 ml screw-cap tubes before DNA extraction. DNA extraction and 16S rRNA Gene Amplification and sequencing protocol are shown in supplementary material protocol S1^23^.

### Processing of 16S sequencing data

Demultiplexed MiSeq FASTQ files were analyzed in QIIME2 using the DADA2 workflow. High read quality is ensured by filtering and trimming reads before processing. The first 5′ 10 bp of all reads were trimmed, and reads truncated on 3' to max 240 and 200 bp for forward and reverse reads, respectively, due to quality dip. Reads with > 2 expected errors under Illumina base model removed. Filtered and trimmed reads are grouped by sequencing run, and the error model fits separately for each run using DADA2 default parameters. Sequence variants were obtained for each run separately using calculated error models and dereplicated input sequences. Sequence variants and counts joined across all runs in the complete sequence table, and de novo chimera removal runs on the entire table^23^.

The final sequence variants taxonomy was assigned to DADA2's RDP classifier using the SILVA database (version 132). Species are identified separately via exact sequence matches (SILVA version 132). Joined with clinical metadata and saved as a phyloseq object for downstream analyses^23^.

### Taxonomic and ecological analysis

A Phyloseq object was used to calculate alpha diversity indexes (i.e., Chao 1, Simpson, Shannon, and Pielou indexes) and β diversity index (Jaccard), computed by R Phyloseq library 1.34.0^52^.

### Supervised machine-learning: explainability analysis approach

To identify bacterial genera associated with different treatments (LM and M), we used the Random Forest algorithm, an ensemble method based on uncorrelated decision trees using the bagging technique. We compared various algorithms (decision tree, logistic regression, naive Bayes, and XGBoost) and selected Random Forest for its predictive performance and interpretability with SHAP values. Python3 (version 3.9.7) with the software library was used for calculations. We labeled data count matrices for M and LM-treated patients as 0 and 1, respectively. 75% of the samples were randomly chosen as the training dataset and the rest as the test dataset. To validate, the Random Forest model was built and evaluated with K-fold cross-validation (n_split = 5) to ensure independent results (Figueroa et al., 2012; Mentch and Hooker, 2016). This involved dividing the data into five equal proportions, using four for training and one for testing each run. Model performance was assessed with the AUC of ROC curves (see Table S5).

Random Forest classifiers that support the (place which) in the main text are reported in the machine learning section at https://github.com/resendislab/Microbiome_two_treatments_Metformine-Linagliptine.

Moreover, we assess the relevance of bacterial genera with Shap values (Shapley additive explanations) using TreeExplainer for the Random Forest algorithm^53^. Shap values use a game-theoretic approach for the best model interpretation and explanation.

Finally, we pairwise compared baseline groups vs both treatments (M and LM) and treatments (M vs LM) at 6 and 12 months of follow-up. SHAP values were used to explore the relevance of the classification process for each genus, quantifying its contribution to classification^53^. Microbiota data faces challenges of technical noise, zero-inflated abundance distribution, and high-dimensionality^54^. However, the random forest model effectively classifies and analyzes microbiome data under these conditions^55^. AUC-ROC was 0.79 and 0.74 for the basal vs M group at 6 and 12 months, respectively, indicating successful training and test data set selection.

### Statistical analyses for 16S metagenomics and their correlations with clinical parameters

#### Statistical analyses for clinical parameters

We estimated the required sample size to observe an effect on GM changes among the treated groups. Briefly, the sample size for this study was determined both a priori and a posteriori using different analytical approaches. In the a priori analysis, we employed an ANOVA (Analysis of Variance) with repeated measures, factoring in two effective groups and three time-point measurements. We aimed for an effect size of 0.25 and set the β error (Type II error) at 20%. This calculation indicated a required sample size of 48 patients. To account for potential dropouts and ensure robustness in our data, we increased this number by 20%, including 167 subjects. For a posteriori analysis, our approach differed slightly. We utilized an ANOVA without repeated measures and considered three effective groups. The effect size was set at 0.01 (eta squared). Under these parameters, the power of our sample was calculated to be 75%. Primary analysis: GM composition change and its relation to IR and insulin secretion in prediabetes patients. The effect of hypoglycemic drugs on IS and β-cells function is analyzed at two levels: (1) changes over follow-up months, and (2) considering drugs and follow-up months. The student's t-test for the first level included independent contrast between basal and 6/12-month groups and dependent contrast between 6 to 12-month subjects. Cohen's d (C-d) and 100-repetition bootstrap 95% confidence intervals were calculated as standardized effect sizes. For the second level, one-way ANOVA was performed, comparing means of clinical parameters for three treatment groups and follow-up months. Two-way ANOVA examined the Time X Treatment impact, and Scheffe's post-hoc was used to identify differences between groups. Logarithmic transformation (base 10) for quantitative variables normalization with bias, and Van der Wader transformation for bacterial abundances.

After machine learning analysis identifying bacteria classifying each group by hypoglycemic drugs and follow-up months, GLMM was performed with IS indices and Pβf as dependent variables. Fixed factors included study group, follow-up month, sex, age, bacteria genera used for classification (Random Forest), and anthropometric parameters (BMI, weight, % body fat, waist circumference). Random factors were stool samples of study subjects. Eta-squared and 95% confidence intervals were calculated with 100 repetitions bootstrap. Interactions (bacteria X Treatment, Bacteria X Time, and Treatment X Time) evaluated. For bidirectional effect, models are executed with diversity and microbial abundance as dependent variables, including study groups, sex, age, IS and Pβf indices, or anthropometric parameters (BMI, weight, % body fat, waist circumference) as fixed factors. Bacteria with significance in both models were considered for subsequent statistical and structural equation modeling (SEM) analysis. Statistical analysis used Stata/SE 16.0, IBM-SPSS version 25, and RStudio version 4.1.1.

### Statistical analysis of 16S metagenomics and its relationship with clinical parameters

To establish GM composition's relationship with IR and insulin secretion in prediabetes patients on hypoglycemic drugs, hierarchical linear regression, GLMM, and structural equation modeling tests were performed. Van der Wader transformation was applied to each bacterial genus for the tests. Statistical analysis was done using Stata/SE 16.0, IBM-SPSS version 25, and RStudio version 4.1.1.

Linear regression was used with IS indices and Pβf as dependent variables. Initially, regression models estimated treatment and follow-up months (by subject) as the main effects and interactions. Subsequently, models were estimated by visiting, sex, and age. Residuals and effect size estimated for each.

The structural equation model (SEM) was used to mediate between BMI, bacterial abundance, significant components, insulin secretion, and IS indices. Coefficients are estimated by a robust method.

Mixed models were used to study hypoglycemic drugs and the effects of new metabolic conditions on bacterial genera. GLMM (Tweedie distribution) was used, with bacterial genera as the dependent variable and hypoglycemic drugs as the independent variable. The model was adjusted for gender, age, BMI, and Matsuda index or Pβf. A stool sample is used as a random effect to consider the within-patient correlation with repeated measures.

## Supplementary Information

Supplementary Information 1.

## Data Availability

The GM datasets generated and/or analyzed during the current study are available in the NCBI repository, [http://www.ncbi.nlm.nih.gov/bioproject/1019716], from the corresponding author, Osbaldo Resendis-Antonio (oresendis047@gmail.com), for gut microbiota data. The clinical datasets used and/or analyzed during the current study are available from the corresponding author, Dr. Rodolfo Guardado-Mendoza (guardamen@gmail.com), upon reasonable request.
