# Author Correction: Effect of metformin and metformin/linagliptin on gut microbiota in patients with prediabetes

**DOI:** 10.1038/s41598-024-72292-4

**Published:** 2024-09-10

**Authors:** Yoscelina Estrella Martínez-López, Daniel Neri-Rosario, Diego Armando Esquivel-Hernández, Cristian Padron-Manrique, Aarón Vázquez-Jiménez, Jean Paul Sánchez-Castañeda, David Girón-Villalobos, Cristian Mendoza-Ortíz, María de Lourdes Reyes-Escogido, Maria Lola Evia-Viscarra, Alberto Aguilar-Garcia, Osbaldo Resendis-Antonio, Rodolfo Guardado-Mendoza

**Affiliations:** 1 Human Systems Biology Laboratory. Instituto Nacional de Medicina Genómica (INMEGEN), México City, Mexico; 2 Programa de Doctorado en Ciencias Médicas, Odontológicas y de la Salud, Universidad Nacional Autónoma de México (UNAM), Ciudad de México, Mexico; 3 Metabolic Research Laboratory, Department of Medicine and Nutrition, University of Guanajuato, León, Guanajuato Mexico; 4 Programa de Maestría en Ciencias Bioquímicas, Universidad Nacional Autónoma de México (UNAM), Ciudad de México, Mexico; 5 Programa de Doctorado en Ciencias Biomédicas, Universidad Nacional Autónoma de México (UNAM), Ciudad de México, Mexico; 6 Endocrinology Department Hospital Regional de Alta Especialidad del Bajío, León, Mexico; 7 Coordinación de la Investigación Científica – Red de Apoyo a la Investigación ‑ Centro de Ciencias de la Complejidad, Universidad Nacional Autónoma de México (UNAM), Ciudad de México, Mexico

Correction to: *Scientific Reports* 10.1038/s41598-024-60081-y, published online 27 April 2024

In the original version of this Article the authors’ given names and surnames were reversed.

The original Article has been corrected.

