# Supplementary material for: Effect of metformin and metformin/linagliptin on gut microbiota in patients with prediabetes
Source: Sci Rep. 2024 Apr 27;14:9678. doi: 10.1038/s41598-024-60081-y (PMC11055900; doi:10.1038/s41598-024-60081-y)
Supplement: Supplementary file 1 — Supplementary Information 1. [file 41598_2024_60081_MOESM1_ESM.docx]

**Effect of metformin and metformin/linagliptin on gut microbiota in patients with prediabetes.**

**Yoscelina Estrella Martínez-López^1,2,3^, Daniel Neri-Rosario^4^, Diego Armando Esquivel-Hernández^1^, Cristian Padron-Manrique**^1,5^**, Aarón Vázquez-Jiménez^1^, Jean Paul Sánchez-Castañeda^4^, David Girón-Villalobos^4^, Cristian Mendoza-Ortíz^4^, María de Lourdes Reyes-Escogido^3^, Osbaldo Resendis-Antonio^1,6*^, Rodolfo Guardado-Mendoza^3*^**

^1^Human Systems Biology Laboratory. Instituto Nacional de Medicina Genómica (INMEGEN). México City, México.

^2^Programa de Doctorado en Ciencias Médicas, Odontológicas y de la Salud, Universidad Nacional Autónoma de México (UNAM). Ciudad de México, México.

^3^Metabolic Research Laboratory, Department of Medicine and Nutrition. University of Guanajuato. León, Guanajuato, México.

^4^Programa de Maestría en Ciencias Bioquímicas, Universidad Nacional Autónoma de México (UNAM). Ciudad de México, México.

^5^Programa de Doctorado en Ciencias Biomédicas, Universidad Nacional Autónoma de México (UNAM). Ciudad de México, México.

^6^Coordinación de la Investigación Científica – Red de Apoyo a la Investigación - Centro de Ciencias de la Complejidad, Universidad Nacional Autónoma de México (UNAM). Ciudad de México, México.

* Corresponding author

| **Variables** | **N** | **Basal** | **N** | **Treatment**  **(6 month)** | **Size effect (Cohen’s D)** | **p-value** |
| --- | --- | --- | --- | --- | --- | --- |
| Age (years) | 65 | 44.08±11.37 | 77 | 48.21±9.85 | 0.356 | 0.0364* |
| SBP (mmHg) | 65 | 123.90±17.93 | 76 | 116.45±15.37 | 0.472 | 0.0060* |
| DBP (mmHg) | 65 | 80.90±11.50 | 76 | 77.02±10.10 | 0.369 | 0.0306* |
| **Anthropometric characteristics** | | | | | | |
| Weight (kg) | 65 | 82.55±18.57 | 78 | 73.49±14.40 | 0.592 | 0.0006* |
| BMI (kg/m^2^) | 65 | 32.02±6.79 | 78 | 27.90±4.55 | 0.776 | 0.0001* |
| Waist circumference (cm) | 61 | 97.64±13.58 | 78 | 89.45±12.80 | 0.547 | 0.0017* |
| Hip circumference (cm) | 61 | 110.57±14.78 | 78 | 102.70±10.33 | 0.669 | 0.0001* |
| Waist/hip ratio | 61 | 0.88±0.08 | 78 | 0.87±0.01 | 0.119 | 0.4859 |
| Body fat (%) | 64 | 38.30±8.81 | 77 | 33.77±8.39 | 0.473 | 0.0195* |
| Visceral fat (AU) | 54 | 9.97±3.99 | 62 | 8.76±3.69 | 0.318 | 0.0900 |
| **Biochemical characteristics** | | | | | | |
| Fasting glucose (mg/dl) | 65 | 108.47±20.72 | 78 | 96.05±9.62 | 1.013 | 0.0001* |
| Glucose 120 min (mg/dl) | 65 | 163.58±46.83 | 77 | 128.60±36.63 | 0.912 | 0.0001* |
| HbA1c (%) | 56 | 5.72±0.79 | 72 | 5.49±0.39 | 0.435 | 0.0160* |
| AUC glucose (OGTT) | 65 | 20569.75±4299.47 | 75 | 16872.64±3710.32 | 1.005 | 0.0145* |
| Matsuda Index | 51 | 2.45±2.03 | 64 | 3.95±3.25 | 0.766 | 0.0001* |
| HOMA-IR | 52 | 4.35±3.94 | 64 | 2.01±2.93 | 1.022 | 0.0001* |
| QUICKI | 51 | 0.32±0.04 | 64 | 0.35±0.04 | 0.738 | 0.0001* |
| HOMA-B | 51 | 106.25±98.13 | 64 | 93.63±136.27 | 0.182 | 0.3348 |
| AUC insulin (OGTT) | 51 | 9216.52±5431.34 | 64 | 7546.25±4982.75 | 0.356 | 0.0605 |
| AIR *(DI0_30/DG0_30)* | 51 | 0.72±0.70 | 64 | 0.95±2.81 | 0.358 | 0.0607 |
| Oral_DI *(AIR*1/insulin)* | 51 | 0.06±0.09 | 64 | 0.11±0.26 | 0.772 | 0.0001* |
| Insulin secretion *(AUCins0-120/AUCgluc0-120)* | 51 | 0.445±0.28 | 64 | 0.448±0.28 | 0.014 | 0.9391 |
| β-Cell function_1_ *(Matsuda*(AUCins_0-120_/AUCgluc_0-120_))* | 51 | 2.63±1.20 | 64 | 5.18±5.08 | 0.956 | 0.0063* |
| β-Cell function_2_ *(Matsuda*(IncAUCins_0-120_/IncAUCgluc_0-120_))* | 51 | 1.09±0.43 | 64 | 1.77±0.71 | 1.113 | 0.0001* |
| Total Cholesterol (mg/dl) | 62 | 182.17±35.08 | 78 | 182.13±35.93 | 0.001 | 0.9941 |
| HDL-c (mg/dl) | 61 | 38.11±11.58 | 76 | 43.97±21.87 | 0.454 | 0.0092* |
| LDL-c (mg/dl) | 48 | 108.09±29.61 | 38 | 103.58±42.39 | 0.112 | 0.6089 |
| VLDL-c (mg/dl) | 47 | 32.52±13.88 | 32 | 33.39±23.09 | 0.059 | 0.7968 |
| Triglycerides (mg/dl) | 62 | 163.32±69.82 | 78 | 147.20±91.85 | 0.224 | 0.1897 |

Independent samples t-test. SBP= systolic blood pressure, DBP= diastolic blood pressure, BMI= body mass index, WC= waist circumference, AC= hip circumference, AUC= area under the curve, IncAUC= increase in area under the curve, OGTT= curve of oral glucose tolerance, AIR= acute insulin response, HOMA-IR= homeostasis model assessment for insulin resistance, HOMA-B= homeostasis model assessment beta-cell, Oral_DI= insulin disposition index, HDL-c= cholesterol high-density lipoprotein cholesterol, LDL-c= low-density lipoprotein cholesterol, VLDL-c= very low-density lipoprotein colesterol, Size effect= Cohen’s D. *p<0.05.

**Table S1.** Contrasts between groups basal vs six months antidiabetic drugs.

| **Variables** | **N** | **Basal** | **N** | **Treatment**  **(12 months)** | **Size effect (Cohen’s D)** | **p-value** |
| --- | --- | --- | --- | --- | --- | --- |
| Age (years) | 65 | 44.08±11.37 | 63 | 47.60±12.11 | 0.265 | 0.1357 |
| SBP (mmHg) | 65 | 123.90±17.93 | 61 | 115.80±15.10 | 0.522 | 0.0040* |
| DBP (mmHg) | 65 | 80.90±11.50 | 61 | 73.64±10.58 | 0.692 | 0.0002* |
| **Anthropometric characteristics** | | | | | | |
| Weight (kg) | 65 | 82.55±18.57 | 63 | 73.95±15.02 | 0.543 | 0.0026* |
| BMI (kg/m^2^) | 65 | 32.02±6.79 | 63 | 28.32±4.35 | 0.702 | 0.0001* |
| Waist circumference (cm) | 61 | 97.64±13.58 | 59 | 90.63±10.49 | 0.582 | 0.0018* |
| Hip circumference (cm) | 61 | 110.57±14.78 | 59 | 103.27±10.64 | 0.599 | 0.0013* |
| Waist/hip ratio | 61 | 0.88±0.08 | 59 | 0.87±0.08 | 0.119 | 0.4859 |
| Body fat (%) | 64 | 38.30±8.81 | 62 | 34.69±7.49 | 0.408 | 0.0241* |
| Visceral fat (AU) | 54 | 9.97±3.99 | 58 | 9.93±3.70 | 0.183 | 0.3346 |
| **Biochemical characteristics** | | | | | | |
| Fasting glucose (mg/dl) | 65 | 108.47±20.72 | 63 | 93.91±9.50 | 1.158 | 0.0001* |
| Glucose 120 min (mg/dl) | 65 | 163.58±46.83 | 63 | 132.18±33.86 | 0.838 | 0.0001* |
| HbA1c (%) | 56 | 5.72±0.79 | 60 | 5.48±0.33 | 0.455 | 0.0158* |
| AUC glucose (OGTT) | 65 | 20569.75±4299.47 | 61 | 17410.17±3023.66 | 0.919 | 0.0001* |
| Matsuda Index | 51 | 2.45±2.03 | 57 | 3.89±3.02 | 0.790 | 0.0001* |
| HOMA-IR | 52 | 4.35±3.94 | 57 | 1.90±1.43 | 1.181 | 0.0001* |
| QUICKI | 51 | 0.32±0.04 | 57 | 0.35±0.04 | 0.854 | 0.0001* |
| HOMA-B | 51 | 106.25±98.13 | 57 | 103.96±135.36 | 0.032 | 0.8665 |
| AUC insulin (OGTT) | 51 | 9216.52±5431.34 | 57 | 7738.98±4775.64 | 0.310 | 0.1111 |
| AIR *(DI0_30/DG0_30)* | 51 | 0.72±0.70 | 57 | 0.84±0.74 | 0.216 | 0.2687 |
| Oral_DI *(AIR*1/insulin)* | 51 | 0.06±0.09 | 57 | 0.10±0.12 | 0.829 | 0.0001* |
| Insulin secretion *(AUCins0-120/AUCgluc0-120)* | 51 | 0.445±0.28 | 57 | 0.444±0.26 | 0.005 | 0.9775 |
| β-Cell function_1_ *(Matsuda*(AUCins_0-120_/AUCgluc_0-120_))* | 51 | 2.63±1.20 | 56 | 4.49±5.51 | 0.779 | 0.0267* |
| β-Cell function_2_ *(Matsuda*(IncAUCins_0-120_/IncAUCgluc_0-120_))* | 51 | 1.09±0.43 | 57 | 1.73±0.66 | 1.056 | 0.0001* |
| Total Cholesterol (mg/dl) | 62 | 182.17±35.08 | 63 | 178.71±36.92 | 0.097 | 0.5897 |
| HDL-c (mg/dl) | 61 | 38.11±11.58 | 63 | 43.13±11.82 | 0.433 | 0.0175* |
| Triglycerides (mg/dl) | 62 | 163.32±69.82 | 63 | 153.09±120.71 | 0.138 | 0.4422 |

Independent samples t-test. SBP= systolic blood pressure, DBP= diastolic blood pressure, BMI= body mass index, WC= waist circumference, AC= hip circumference, AUC= area under the curve, IncAUC= increase in area under the curve, OGTT= curve of oral glucose tolerance, AIR= acute insulin response, HOMA-IR= homeostasis model assessment for insulin resistance, HOMA-B= homeostasis model assessment beta-cell, Oral_DI= insulin disposition index, HDL-c= cholesterol high-density lipoprotein cholesterol, LDL-c= low-density lipoprotein cholesterol, VLDL-c= very low-density lipoprotein colesterol, Size effect= Cohen’s D. *p<0.05.

**Table S2.** Contrasts between groups basal vs 12 months antidiabetic drugs.

| **Variables** | **N** | **Treatment**  **(6 months)** | **N** | **Treatment**  **(12 months)** | **Size effect**  **(Δ de Glass)** | **p-value** |
| --- | --- | --- | --- | --- | --- | --- |
| SBP (mmHg) | 24 | 118.96±15.54 | 24 | 114.86±14.67 | 0.273 | 0.1664 |
| DBP (mmHg) | 24 | 78.06±10.53 | 24 | 73.83±8.84 | 0.420 | 0.0223* |
| **Anthropometric characteristics** | | | | | | |
| Weight (kg) | 24 | 72.27±14.85 | 24 | 72.18±18.86 | 0.253 | 0.8763 |
| BMI (kg/m^2^) | 24 | 27.66±3.04 | 24 | 27.69±2.95 | 0.010 | 0.9032 |
| Waist circumference (cm) | 23 | 89.72±9.80 | 23 | 89.45±9.32 | 0.034 | 0.7829 |
| Hip circumference (cm) | 23 | 100.71±6.93 | 23 | 101.43±6.73 | 0.102 | 0.5330 |
| Waist/hip ratio | 23 | 0.889±0.11 | 23 | 0.881±0.09 | 0.082 | 0.5318 |
| Body fat (%) | 22 | 35.56±6.57 | 22 | 35.29±5.74 | 0.062 | 0.6934 |
| Visceral fat (AU) | 14 | 7.87±2.36 | 14 | 8.70±2.77 | 0.394 | 0.0654 |
| **Biochemical characteristics** | | | | | | |
| Fasting glucose (mg/dl) | 24 | 92.5±6.05 | 24 | 93.13±6.44 | 0.104 | 0.5825 |
| Glucose 120 min (mg/dl) | 24 | 122.80±30.58 | 24 | 122.73±32.78 | 0.020 | 0.9926 |
| HbA1c (%) | 22 | 5.43±0.21 | 22 | 5.49±0.21 | 0.286 | 0.3113 |
| AUC glucose (OGTT) | 22 | 16006.71±2872.06 | 22 | 16988.69±2413.02 | 0.320 | 0.0507 |
| Matsuda Index | 21 | 3.88±3.66 | 21 | 3.73±2.37 | 0.194 | 0.7427 |
| HOMA-IR | 21 | 2.08±4.40 | 21 | 1.99±1.16 | 0.227 | 0.8113 |
| QUICKI | 21 | 0.344±0.04 | 21 | 0.345±0.03 | 0.025 | 0.9493 |
| HOMA-B | 21 | 113.37±208.46 | 21 | 110.02±66.78 | 0.165 | 0.8660 |
| AUC insulin (OGTT) | 21 | 8160.34±4878.59 | 21 | 8688.30±4309.54 | 0.023 | 0.4757 |
| AIR *(DI0_30/DG0_30)* | 21 | 1.07±0.85 | 21 | 0.85±0.52 | 0.459 | 0.2091 |
| Oral_DI *(AIR*1/insulin)* | 21 | 0.12±0.14 | 21 | 0.10±0.05 | 0.429 | 0.5128 |
| Insulin secretion *(AUCins0-120/AUCgluc0-120)* | 21 | 0.52±0.28 | 21 | 0.51±0.24 | 0.107 | 0.8595 |
| β-Cell function_1_ *(Matsuda*(AUCins_0-120_/AUCgluc_0-120_))* | 21 | 6.33±7.20 | 21 | 5.23±3.82 | 0.153 | 0.2626 |
| β-Cell function_2_ *(Matsuda*(IncAUCins_0-120_/IncAUCgluc_0-120_))* | 21 | 2.03±0.81 | 21 | 1.92±0.45 | 0.247 | 0.5084 |
| Total Cholesterol (mg/dl) | 24 | 184.93±40.81 | 24 | 176.25±33.66 | 0.233 | 0.2448 |
| HDL-c (mg/dl) | 22 | 49.45±21.87 | 22 | 46.72±12.01 | 0.058 | 0.6573 |
| Triglycerides (mg/dl) | 24 | 144.45±123.59 | 24 | 160.58±165.25 | 0.144 | 0.2661 |

Dependent samples t-test. SBP= systolic blood pressure, DBP= diastolic blood pressure, BMI= body mass index, WC= waist circumference, AC= hip circumference, AUC= area under the curve, IncAUC= increase in area under the curve, OGTT= curve of oral glucose tolerance, AIR= acute insulin response, HOMA-IR= homeostasis model assessment for insulin resistance, HOMA-B= homeostasis model assessment beta-cell, Oral_DI= insulin disposition index, HDL-c= cholesterol high-density lipoprotein cholesterol, LDL-c= low-density lipoprotein cholesterol, VLDL-c= very low-density lipoprotein colesterol, Size effect= Δ de Glass. *p<0.05.

**Table S3.** Contrasts between groups with pharmacological treatment (6 vs 12 months follow-up).

| **Variables** | **N** | **Basal** | **N** | **Metformin**  **(6 months)** | **N** | **Metformin**  **(12 months)** | **N** | **Linagliptin/Metformin**  **(6 months)** | **N** | **Linagliptin/metformin**  **(12 months)** | **p-value** |
| --- | --- | --- | --- | --- | --- | --- | --- | --- | --- | --- | --- |
| Age (years) | 65 | 44.08±11.37^c^ | 44 | 47.05±10.89^b,c^ | 28 | 44.47±12.82^c^ | 33 | 49.79±8.33ª | 35 | 50.26±11.26ª | 0.089 |
| SBP (mmHg) | 65 | 123.90±17.93ª | 44 | 114.00±14.20^b^ | 28 | 116.93±14.59^b^ | 32 | 119.91±16.41ª | 33 | 114.85±15.69^b^ | 0.008 |
| DBP (mmHg) | 65 | 80.90±11.50ª | 44 | 75.09±9.03^b,c^ | 28 | 75.44±8.48^b,c^ | 32 | 79.76±10.94ª | 33 | 71.65±12.06^c^ | 0.0001 |
| **Anthropometric characteristics** | | | | | | | | | | |  |
| Weight (kg) | 65 | 82.55±18.57ª | 44 | 71.49±13.22^b,c^ | 28 | 76.91±17.78ª | 33 | 76.15±15.56^b,c^ | 35 | 71.67±11.98^c^ | 0.001 |
| BMI (kg/m^2^) | 65 | 32.02±6.79ª | 44 | 27.11±4.28^c^ | 28 | 29.56±4.98^b^ | 33 | 29.01±4.71^b^ | 35 | 27.36±3.51^b,c^ | 0.0001 |
| WC (cm) | 61 | 97.64±13.58ª | 44 | 86.84±13.69^b^ | 28 | 92.99±11.52ª | 33 | 92.95±11.05ª | 31 | 88.55±9.08^b^ | 0.001 |
| Body fat (%) | 64 | 38.30±8.81ª | 44 | 31.81±8.20c | 27 | 36.37±6.89ª^,b^ | 33 | 36.57±8.08^a,b^ | 35 | 33.44±7.83^b,c^ | 0.003 |
| Visceral fat (AU) | 54 | 9.97±3.99 | 42 | 8.39±3.73 | 25 | 9.68±4.47 | 20 | 9.62±3.60 | 33 | 8.96±2.98 | 0.275 |
| **Biochemical characteristics** | | | | | | | | | | |  |
| Matsuda Index | 51 | 2.45±2.03^c^ | 30 | 4.96±2.57^b^ | 24 | 5.22±3.57ª^,b^ | 33 | 4.74±0.378^b^ | 33 | 4.17±2.51^b^ | 0.0001 |
| HOMA-IR | 52 | 4.35±3.94ª | 30 | 2.45±3.54^b^ | 24 | 2.28±1.53^b^ | 33 | 3.03±2.29^b^ | 33 | 2.33±1.37^b^ | 0.0001 |
| AIR *(DI0_30/DG0_30)* | 51 | 0.72±0.70^b^ | 30 | 0.88±0.53^b^ | 24 | 0.95±0.72^b^ | 33 | 1.95±3.79ª | 33 | 1.05±0.76^b^ | 0.023 |
| Oral_DI *(AIR*1/insulin)* | 51 | 0.06±0.09^c^ | 30 | 0.13±0.08^b^ | 24 | 0.08±0.17^b^ | 33 | 0.21±0.35ª^,b^ | 33 | 0.11±0.06^b^ | 0.0001 |
| Insulin secretion *(AUCins0-120/AUCgluc0-120)* | 51 | 0.445±0.28^b^ | 30 | 0.41±0.17^b^ | 24 | 0.42±0.21^b^ | 33 | 0.61±0.33ª | 33 | 0.57±0.28ª | 0.029 |
| β-Cell function_1_ *(Matsuda*(AUCins_0-120_/AUCgluc_0-120_))* | 51 | 2.63±1.20^c^ | 30 | 5.45±3.67^b^ | 24 | 6.20±8.01^b^ | 33 | 7.64±5.93ª | 33 | 5.27±2.56^b^ | 0.018 |
| β-Cell function_2_ *(Matsuda*(IncAUCins_0-120_/IncAUCgluc_0-120_))* | 51 | 1.09±0.43^c^ | 30 | 1.78±0.60^b^ | 24 | 1.75±0.85^b^ | 33 | 2.03±0.79ª | 33 | 1.92±0.49^b^ | 0.0001 |

SBP= systolic blood pressure, DBP= diastolic blood pressure, BMI= body mass index, WC= waist circumference, AC= hip circumference, AUC= area under the curve, IncAUC= increase in area under the curve, OGTT= curve of oral glucose tolerance, AIR= acute insulin response, HOMA-IR= homeostasis model assessment for insulin resistance, Oral_DI= insulin disposition index.

**Table S4.** The contrast between groups.


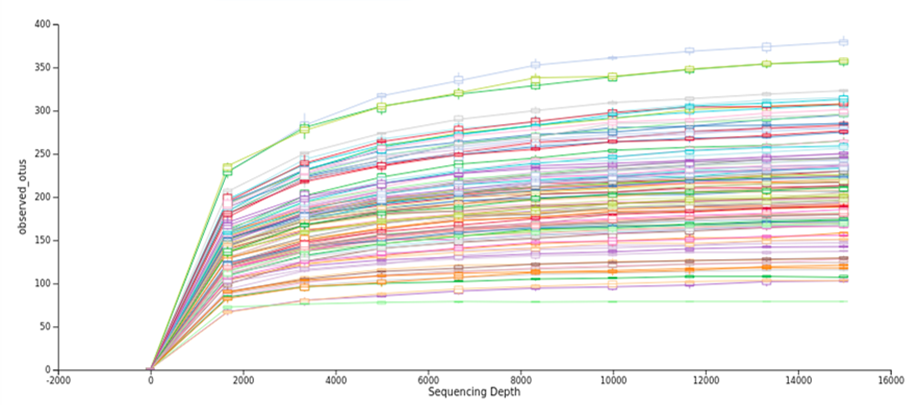


**Figure S1.** Rarefaction curve.

**
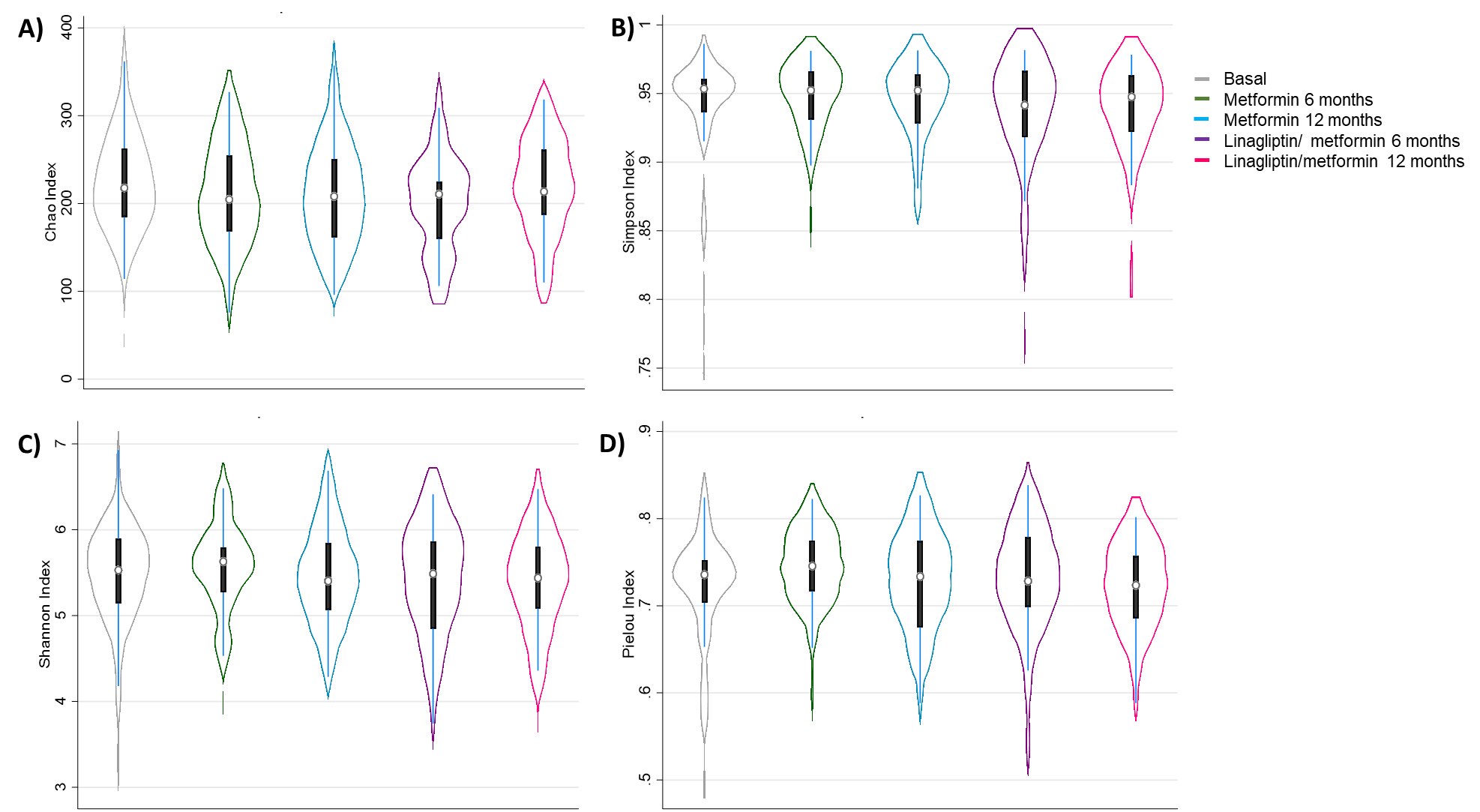
**

**Figure S2.** The microbial community richness (Chao 1 index; A, p = 0.550 and Simpson index; B, p = 0.817) and diversity (Shannon index; C, p = 0.796 and Pielou index; D, p = 0.469). Violin plots show the median, quartiles, and min/max values. Kruskal-Wallis test *p < 0.05.

**
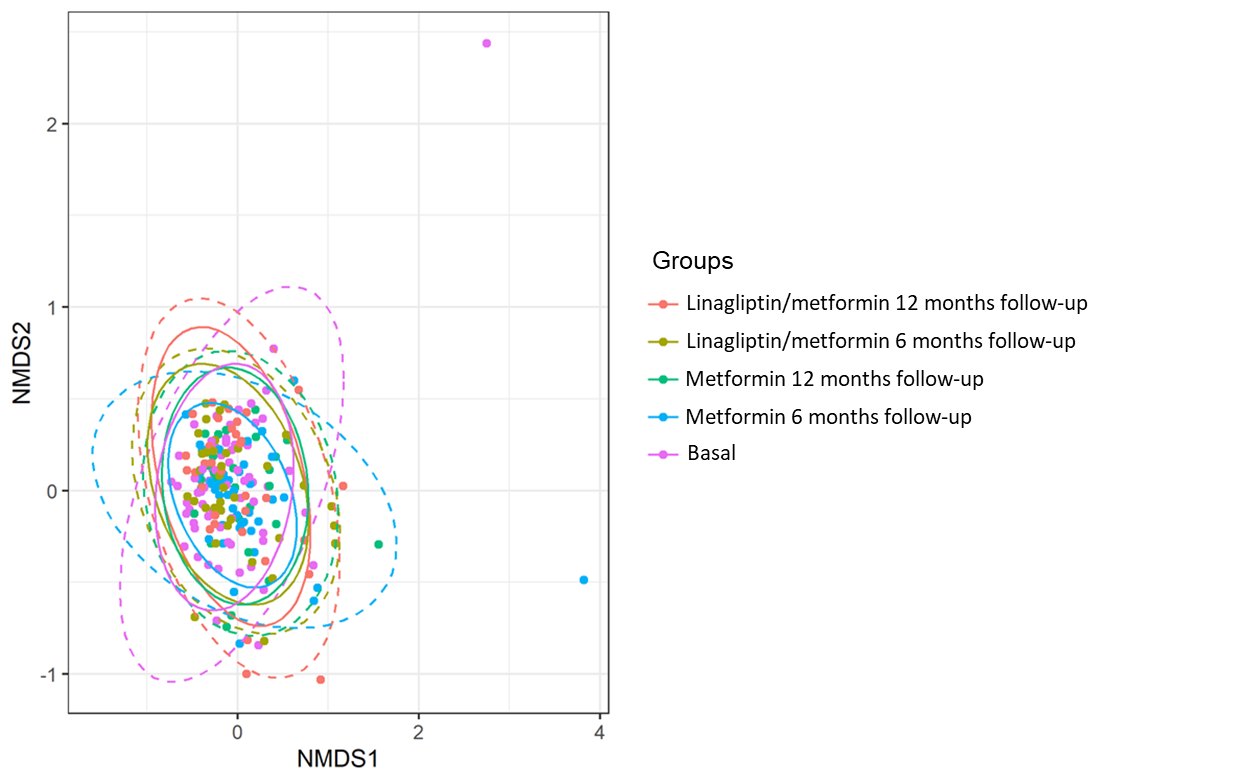
**

**Figure S3.** Beta diversity analysis. The beta diversity analysis with Jaccard distances shows that we cannot define any grouping pattern from the microbial diversity between the analysis groups.


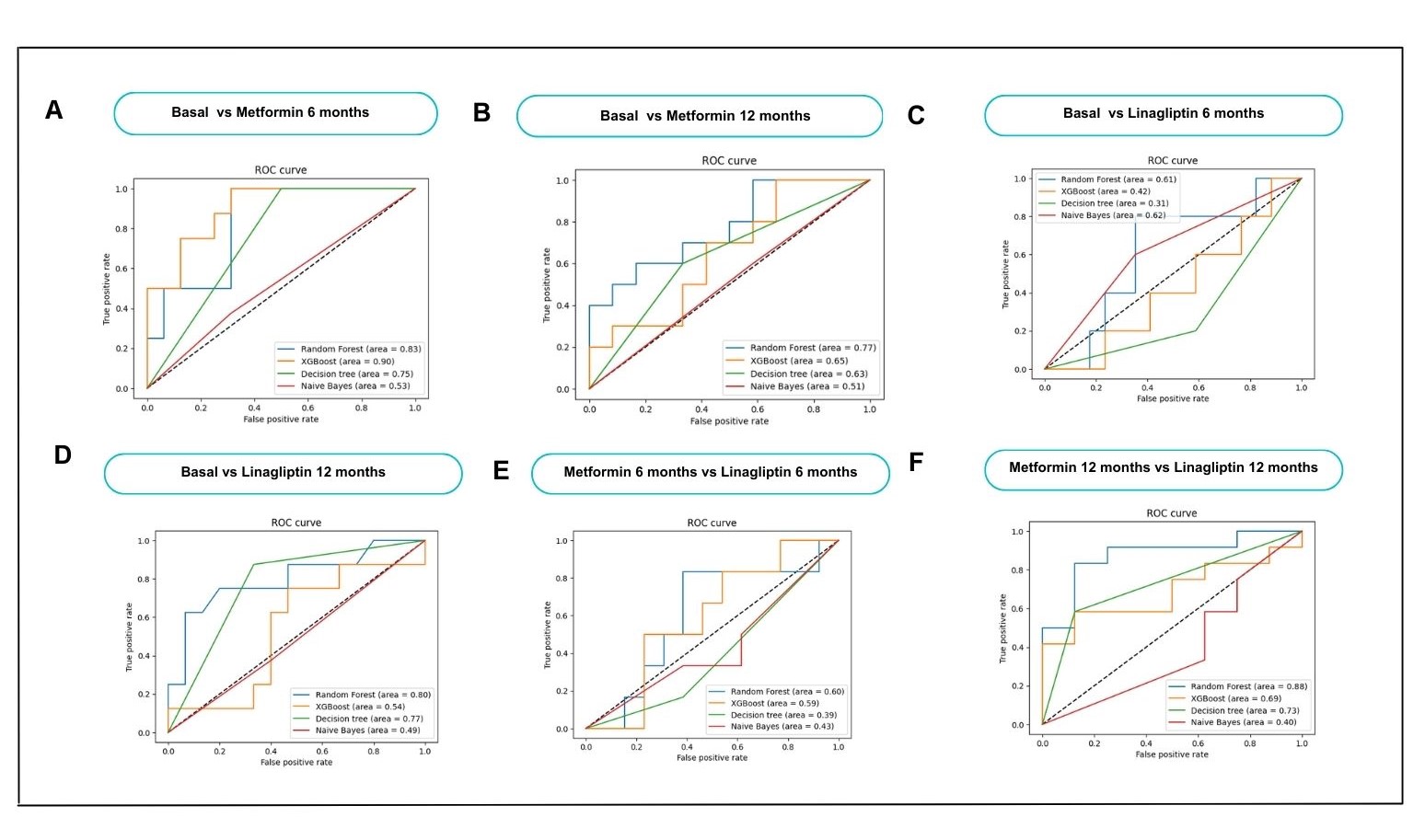


**Figure S4.** Comparison analysis between models. We compared the predictive performance using area under the curve (AUC) of the ROC curves of different algorithms to select the method to use, including the decision tree, naive Bayes, and XGBoost.

| **Group** | **Accuracy** | **AUC** | **Mean Accuracy** | **SD (CV=5)** | **Mean**  **AUC** | **DE (CV=5)** |
| --- | --- | --- | --- | --- | --- | --- |
| **Basal vs Metformin 6 months** | 0.66 | 0.75 | 0.69 | 0.02 | 0.79 | 0.04 |
| **Basal vs Linagliptin/Metformin 6 months** | 0.59 | 0.66 | 0.65 | 0.13 | 0.60 | 0.12 |
| **Basal vs Metformin 12 months** | 0.59 | 0.60 | 0.68 | 0.07 | 0.74 | 0.07 |
| **Basal vs Linagliptin/Metformin 12 months** | 0.73 | 0.74 | 0.65 | 0.4 | 0.66 | 0.13 |
| **Metformin 6 months vs Linagliptin/Metformin 6 months** | 0.73 | 0.76 | 0.63 | 0.15 | 0.66 | 0.18 |
| **Metformin 12 months vs Linagliptin/Metformin 12 months** | 0.75 | 0.75 | 0.65 | 0.1 | 0.74 | 0.11 |

**Table S5.** Machine Learning algorithms performance. We compare the performance of comparisons by pharmacological treatment and monitoring month using precision and AUC values. We use the stratified cross-validation technique to obtain the standard deviation in our results (K fold = 10).

| **Fixed effects** | **β** | **SE** | **p-value** | **95% CI** | | **Eta-Squared** | **95%CI** | |
| --- | --- | --- | --- | --- | --- | --- | --- | --- |
| ***Butyrycicoccus*** | | | | | | | | |
| *Matsuda Index* | 0.666 | 0.337 | 0.048 | 0.006 | 1.326 | 0.027 | 0.0001 | 0.099 |
| *AUCinsgluc_OGTT* | -0.717 | 0.359 | 0.046 | -1.421 | -0.012 | 0.027 | 0.0001 | 0.101 |
| ***Erysipelotrichaceae UCG-003*** | | | | | | | | |
| *AUCinsgluc_OGTT* | -0.824 | 0.338 | 0.015 | -1.487 | -0.162 | 0.039 | 0.0006 | 0.121 |
| ***[Eubacterium] xylanophilum group*** | | | | | | | | |
| *Matsuda Index* | -0.630 | 0.304 | 0.038 | -1.227 | -0.033 | 0.029 | 0.0001 | 0.104 |
| *AUCinsgluc_OGTT* | 0.647 | 0.325 | 0.047 | 0.009 | 1.285 | 0.027 | 0.0001 | 0.100 |
| ***Granulicatella*** | | | | | | | | |
| *Matsuda Index* | 0.544 | 0.241 | 0.024 | 0.072 | 1.017 | 0.034 | 0.0001 | 0.113 |
| ***Ruminococcaceae UCG-008*** | | | | | | | | |
| *Matsuda Index* | -0.366 | 0.185 | 0.042 | -0.729 | -0.004 | 0.027 | 0.0001 | 0.099 |
| ***Prevotella 6*** | | | | | | | | |
| *Matsuda Index* | -0.480 | 0.223 | 0.031 | -0.916 | -0.044 | 0.031 | 0.0001 | 0.108 |
| *HOMA-IR* | 0.385 | 0.188 | 0.041 | 0.017 | 0.754 | 0.028 | 0.0001 | 0.103 |
| ***Catabacter*** | | | | | | | | |
| *Matsuda Index* | -0.508 | 0.232 | 0.029 | -0.964 | -0.053 | 0.032 | 0.0001 | 0.110 |
| *HOMA-IR* | 0.390 | 0.196 | 0.047 | 0.005 | 0.776 | 0.027 | 0.0001 | 0.100 |
| ***Actinomyces*** | | | | | | | | |
| *HOMA-IR* | -0.752 | 0.270 | 0.005 | -1.279 | -0.223 | 0.052 | 0.0036 | 0.139 |
| *ORAL-DI* | 0.551 | 0.249 | 0.027 | 0.063 | 1.038 | 0.034 | 0.0001 | 0.113 |
| ***Negativibacillus*** | | | | | | | | |
| *HOMA-IR* | -0.450 | 0.227 | 0.048 | -0.895 | -0.005 | 0.027 | 0.0001 | 0.100 |
| *ORAL-DI* | 0.537 | 0.204 | 0.008 | 0.138 | 0.936 | 0.047 | 0.0022 | 0.134 |
| *DIsp_Index2* | 0.972 | 0.349 | 0.005 | 0.289 | 1.655 | 0.052 | 0.0036 | 0.139 |
| ***Bilophila*** | | | | | | | | |
| *AIR* | -0.566 | 0.240 | 0.018 | -1.036 | -0.096 | 0.038 | 0.0001 | 0.119 |
| ***Mogibacterium*** | | | | | | | | |
| *AIR* | 0.652 | 0.222 | 0.003 | 0.216 | 1.088 | 0.057 | 0.0052 | 0.148 |
| *ORAL-DI* | 0.630 | 0.205 | 0.002 | 0.227 | 1.031 | 0.063 | 0.0069 | 0.156 |
| ***Lachnospiraceae UCG-004*** | | | | | | | | |
| *AIR* | -0.423 | 0.199 | 0.034 | -0.814 | -0.033 | 0.031 | 0.0001 | 0.108 |
| *ORAL-DI* | -0.497 | 0.182 | 0.006 | -0.854 | -0.139 | 0.050 | 0.0030 | 0.138 |
| *Disp_Index2* | -1.069 | 0.311 | 0.001 | -1.679 | -0.461 | 0.077 | 0.0127 | 0.173 |
| *AUCinsgluc_OGTT* | -0.531 | 0.259 | 0.040 | -1.039 | -0.023 | 0.029 | 0.0001 | 0.103 |
| ***Fournierella*** | | | | | | | | |
| *AIR* | 0.448 | 0.214 | 0.036 | 0.029 | 0.867 | 0.030 | 0.0001 | 0.107 |
| ***Atopobium*** | | | | | | | | |
| *ORAL-DI* | 0.508 | 0.209 | 0.015 | 0.098 | 0.918 | 0.404 | 0.0006 | 0.123 |
| *Disp_Index2* | 0.787 | 0.358 | 0.028 | 0.086 | 1.489 | 0.033 | 0.0001 | 0.110 |
| ***Catenibacterium*** | | | | | | | | |
| *Disp_Index2* | 0.785 | 0.301 | 0.009 | 0.194 | 1.375 | 0.045 | 0.0019 | 0.130 |
| ***Eggerthellaceae uncultured*** | | | | | | | | |
| *AUCinsgluc_OGTT* | 0.571 | 0.262 | 0.030 | 0.056 | 1.085 | 0.032 | 0.0001 | 0.109 |
| ***Peptococcaceae unclutured*** | | | | | | | | |
| *AUCinsgluc_OGTT* | 0.618 | 0.302 | 0.041 | 0.027 | 1.208 | 0.028 | 0.0001 | 0.103 |
| N | 142 | | | | |  |  |  |

**Table S6.** MLGM for bacterial genera and effect size and confidence interval for each bacterium.

| **Fixed effects** | **β** | **SE** | **p-value** | **95% CI** | | **Eta-Squared** | **95%CI** | |
| --- | --- | --- | --- | --- | --- | --- | --- | --- |
| ***Catabacter*** | | | | | | | | |
| V6 Tmet | -0.327 | 0.194 | 0.093 | -0.708 | 0.054 | 0.043 | 0.0001 | 0.101 |
| V12 Tmet | -0.278 | 0.177 | 0.117 | -0.625 | 0.069 |  |  |  |
| V6 Tlina+met | -0.166 | 0.187 | 0.372 | -0.532 | 0.199 |  |  |  |
| V12 Tlina+met | 0.049 | 0.169 | 0.770 | -0.282 | 0.380 |  |  |  |
| Sexo | 0.133 | 0.121 | 0.269 | -0.103 | 0.369 | 0.008 | 0.0001 | 0.063 |
| Edad | 0.038 | 0.485 | 0.937 | -0.912 | 0.988 | 0.00004 | 0.0001 | 0.013 |
| IMC | -0.423 | 0.797 | 0.596 | -1.984 | 1.139 | 0.0019 | 0.0001 | 0.042 |
| Matsuda Index | -0.503 | 0.233 | 0.031 | -0.959 | -0.047 | 0.031 | 0.0001 | 0.108 |
| N | 143 |  |  |  |  |  |  |  |

**Table S7.** Mixed generalized linear models for the genus *Catabacter* and each bacterium's effect size and confidence interval.

| **Fixed effects** | **β** | **SE** | **p-value** | **95% CI** | | **Eta-Squared** | **95%CI** | |
| --- | --- | --- | --- | --- | --- | --- | --- | --- |
| ***Lachnospiraceae UCG-004*** | | | | | | | | |
| V6 Tmet | 0.467 | 0.205 | 0.023 | 0.065 | 0.870 | 0.059 | 0.0001 | 0.126 |
| V12 Tmet | 0.175 | 0.183 | 0.339 | -0.184 | 0.533 |  |  |  |
| V6 Tlina+met | 0.519 | 0.202 | 0.010 | 0.123 | 0.914 |  |  |  |
| V12 Tlina+met | 0.206 | 0.187 | 0.268 | -0.160 | 0.573 |  |  |  |
| Sexo | 0.193 | 0.125 | 0.125 | -0.053 | 0.438 | 0.016 | 0.0007 | 0.081 |
| Edad | -0.695 | 0.503 | 0.167 | -1.679 | 0.290 | 0.013 | 0.0001 | 0.074 |
| IMC | 0.222 | 0.794 | 0.780 | -1.335 | 1.779 | 0.0005 | 0.0001 | 0.031 |
| Disp_Index2 | -0.990 | 0.317 | 0.002 | -1.612 | -0.368 | 0.064 | 0.0076 | 0.156 |
| N | 166 |  |  |  |  |  |  |  |

**Table S8.** Mixed generalized linear models for the genus *Lachnospiraceae UCG-004* and effect size and confidence interval for each bacterium.

# Protocol S1. Fecal sample processing protocol.

## DNA Extraction

DNA extraction used MoBio PowerSoil DNA Isolation kit (Mo Bio Laboratories, Inc. Carlsbad, USA) following the manufacturer’s instructions with modifications. After adding C1 solution and mixing, 25 µl proteinase K solution was added, vortexed, and incubated at 65°C for 10 min, with inversion mixing every three min. Tubes were secured horizontally and vortexed at 3,000 rpm for 15 min. Incubated at 95°C for 10 min with mixing as mentioned. Total DNA eluted in 100 µl sterile water. DNA concentration quantified with Qubit (Thermo Scientific, USA) and validated by Nanodrop (ND 2000, Thermo Scientific, USA).

## 16S rRNA Gene Amplification and Sequencing

DNA templates used in a two-step PCR to sequence the V4 hypervariable region of bacterial 16S rRNA gene. Fusion primers contained v4 region and Nextera Illumina adapter sequences for multiplexing pooled libraries. Initial PCR used primers with partial Nextera adapter and V4 targeting forward or reverse primer sequence in agreement with.

NEXT_16S_V4_U515_F

5’-TCGTCGGCAGCGTCAGATGTGTATAAGAGACAGGTGCCAGCMGCCGCGGTAA-3′

NEXT_16S_V4_E786_R

5’-GTCTCGTGGGCTCGGAGATGTGTATAAGAGACAGGGACTACHVGGGTWTCTAAT-3′

For each sample, an equal DNA template (up to 12.5 ng per reaction) is used. Reactions had 3 min denature step at 94°C, followed by 25 cycles of denaturation at 94°C for 45 s, annealing at 50°C for 60 s, and extension at 72°C for 90 s, with final extension at 72°C for 10 min. All reactions used 2x KAPA HiFi HotStart ReadyMix to generate amplicons^[52](https://paperpile.com/c/ubt0pf/IcTN)^.

Amplicons purified using Agencourt Ampure XP beads (Beckman Coulter) at 1.25x (v/v) proportion. PCR products checked via electrophoresis in 2% (w/v) agarose gels in TAE buffer stained with SYBR Gold and visualized under UV light.

For each amplicon, a second PCR performed at 95°C for 3 min, followed by 8 cycles of denaturation at 95°C for 30 s, annealing at 55°C for 30 s, and extension at 72°C for 30 s, with final extension at 72°C for 5 min. 5 µl of previous purified DNA template and primers attaching dual indices and Illumina sequencing adapters from Nextera XT kit used. PCR products purified as before, DNA concentration determined using Qubit® 2.0 Broad Range Assay (Life Technologies™). Agilent TapeStation (Agilent, Santa Clara, CA) with DNA High Sensitivity kit used for PCR product size verification, only for 23 amplicons. Samples randomly distributed into five pools in similar proportions, then mixed equally (to 10 nM). Final concentration of each pool determined using Qubit® 2.0.

Pools diluted to 9 pM for sequencing using 2x250 bp paired-end chemistry v2 on Illumina MiSeq. All samples distributed in five pools equally (to 10 nM) as per consecutive numbers assigned by the experimental lab. Final concentration of each pool determined using Qubit® 2.0. Amplicons denatured with 0.2 N NaOH, further diluted according to MiSeq user guide, combined with denatured PhiX control library. PhiX spiked into the amplicon pool at 10% relative concentration. Image processing and base calling done on BaseSpace cloud from Illumina (http://basespace.illumina.com).
